# Multi-omics mapping of PSC genetic risk to a high TWAS-active JAML+ lipid-associated macrophage program: bridging single-cell heterogeneity, spatial fibrotic niches, and machine learning selection

**DOI:** 10.3389/fcell.2026.1923026

**Published:** 2026-09-04

**Authors:** Zhongyan Du, Zhihao Xu, Chenxiao Yang, Yuanyuan Zhang, Quan Jiang, Xiaolan Wang

**Affiliations:** 1 Department of Hepatobiliary Surgery, The First Affiliated Hospital of Jinan University, Huaqiao Hospital, Guangzhou, China; 2 Department of Pathophysiology, Key Laboratory of State Administration of Traditional Chinese Medicine, School of Medicine, Jinan University, Guangzhou, China; 3 Urban Vocational College of Sichuan, Chengdu, China

**Keywords:** lipid-associated macrophages, machine learning, primary sclerosing cholangitis (PSC), scRNA-seq, spatial transcriptomics, TWAS, virtual knockout

## Abstract

**Background:**

Primary sclerosing cholangitis (PSC) is a chronic cholestatic liver disease with progressive peribiliary inflammation and fibrosis. Disease-modifying therapies are lacking, and the cell-type-specific mechanisms linking genetic susceptibility to pathogenic immune states remain incompletely understood.

**Methods:**

We performed a transcriptome-wide association study (TWAS) using PSC GWAS summary statistics and GTEx v8 liver eQTL weights. TWAS-prioritized genes were mapped onto a PSC liver single-cell RNA-seq atlas (GSE247128) using integrated gene set scoring (irGSEA). Monocyte/macrophage subsets were re-clustered and lipid-associated macrophages (LAMs) were stratified by TWAS activity. Core genes distinguishing high-versus low-activity LAMs were identified using seven machine-learning feature selection algorithms. JAML was validated in bulk transcriptomic cohorts (GSE119600, GSE177044), evaluated by immune infiltration analysis, examined by cell–cell communication inference (CellChat), assessed by virtual knockout (scTenifoldKnk), and spatially localized using Visium FFPE spatial transcriptomics (PSC: GSE245620; control: GSE240429), and validated at the protein level using Western blot and ELISA in an *in vitro* macrophage model.

**Results:**

TWAS activity was predominantly enriched in the monocyte/macrophage lineage. Within this lineage, LAMs (TREM2+ GPNMB+ APOC1+) showed the highest TWAS activity and occupied late pseudotime states. High-activity LAMs were enriched in fibrosis-related pathways (e.g., TGF-β, NOTCH, WNT/β-catenin) and innate immune pathways (e.g., TLR2/4-MAPK, NLRP3 inflammasome). Multi-algorithm feature selection identified JAML (AMICA1) as a core discriminator of high-versus low-activity LAMs. JAML was upregulated in PSC in bulk cohorts, associated with higher macrophage and lower activated CD8^+^ T-cell infiltration, showed enhanced cell–cell communication signatures, and spatially co-localized with LAM and fibrosis scores in PSC tissue sections. *In vitro*, JAML upregulation accompanied NF-κB p65 phosphorylation and PD-L1 expression in LPS/IL-6-stimulated macrophages, and JAML knockdown attenuated these responses.

**Conclusion:**

Integrating TWAS with single-cell and spatial transcriptomics highlights a genetically linked, highly active LAM state in PSC and nominates JAML + LAMs as a testable effector population potentially connecting genetic susceptibility to peribiliary fibrosis, with *in vitro* evidence supporting a JAML/NF-κB/PD-L1 inflammatory axis.

## Introduction

Primary sclerosing cholangitis (PSC) is a chronic cholestatic liver disease characterized by progressive inflammation, fibrosis, and multifocal strictures of the intra- and extrahepatic bile ducts (Europen Association for the Study of the Liver, 2022; Dyoson et al., 2023). Typical features include periductal “onion-skin” (concentric periductal) fibrosis, a histological hallmark of PSC (Cançado and Hirschfield, 2024), together with cholangiocyte injury and associated immune-inflammatory responses (Jalan-Sakrikar et al., 2025). Disease progression can lead to biliary cirrhosis and markedly increases the risk of cholangiocarcinoma (Lim, 2023). Because early symptoms are often non-specific and sensitive non-invasive biomarkers are lacking, many patients already present with biliary strictures or advanced fibrosis at diagnosis (Dyson et al., 2023; Tabibian et al., 2018). Hence, non-invasive diagnostic and prognostic tools are urgently needed for PSC.

Cholangiocyte injury and the subsequent immune-inflammatory reaction are major drivers of PSC initiation and progression (Jalan-Sakrikar et al., 2025; Karlsen et al., 2017). Emerging single-cell studies in liver fibrosis have shown that macrophages play a central role in shaping fibrotic microenvironments. Fabre et al. identified a pro-fibrogenic macrophage subset marked by TREM2, GPNMB, SPP1, and CD9, which expands in fibrotic tissues and differentiates from circulating monocytes (Fabre et al., 2023). Ramachandran et al. further revealed that scar-associated macrophages are linked to pro-fibrogenic pathways such as NOTCH and PDGFR and display antigen-presenting features, including high expression of MHC class II and costimulatory molecules (Ramachandran et al., 2019). However, how immune cell heterogeneity in PSC relates to genetic susceptibility remains largely unknown.

Single-cell RNA sequencing (scRNA-seq) provides a powerful approach to dissect cellular composition and state heterogeneity in complex diseases (Hao et al., 2013; Korsunsky et al., 2019). Nevertheless, cell-type-resolved mapping of genetic risk signals and their putative effector cells in PSC is still limited. Transcriptome-wide association studies (TWAS) integrate expression quantitative trait locus (eQTL) information to test associations between genetically predicted gene expression and disease phenotypes, thereby facilitating functional interpretation of GWAS signals (Mai et al., 2023; Gusev et al., 2016). Compared with GWAS, TWAS aggregates multiple variants into gene-level associations and can help prioritize candidate genes. Combining TWAS with scRNA-seq offers a strategy to characterize genetic risk-related transcriptional activity at single-cell resolution, identify candidate pathogenic cell subsets and molecular targets, and support biomarker development.

In this study, we first performed TWAS to identify candidate genes whose genetically predicted expression levels were associated with PSC. We then constructed a single-cell atlas of PSC liver and mapped TWAS activity across distinct cell types and subsets to identify cell populations enriched for genetic risk-related signals. Using multiple machine learning algorithms (LASSO, random forest, XGBoost, Boruta, GBM, ABESS, and decision tree), we extracted core signature genes from the high-activity macrophage subset and focused on JAML for further investigation. Spatial transcriptomics was subsequently applied to characterize the spatial association of JAML expression with lipid-associated macrophages and fibrosis-related features in PSC tissues. Furthermore, *in vitro* functional experiments using inflammatory macrophage models were performed to investigate the involvement of JAML in NF-κB activation, PD-L1 expression, and IL-6 secretion. Collectively, this study integrates genetic association, single-cell heterogeneity, spatial organization, and experimental validation to provide insights into the potential role of JAML-positive macrophages in PSC pathogenesis and to identify candidate biomarkers for further translational investigation.

## Methods

### Data collection

A total of 16 single-cell RNA sequencing (scRNA-seq) samples, including 8 PSC samples, 6 CONTROL samples, and 2 PBC samples, were obtained from the GEO database (https://www.ncbi.nlm.nih.gov/geo/) under accession number GSE247128. Two bulk RNA-seq datasets were also retrieved from GEO: GSE119600 (47 normal and 45 PSC samples) and GSE177044 (78 normal and 42 PSC samples). For TWAS analysis, we used genome-wide association study (GWAS) summary statistics from the International PSC Study Group (IPSCSG) under GWAS Catalog accession number GCST004030, comprising 14,890 individuals (2,871 PSC cases and 12,019 controls).

### Bulk RNA-seq data preprocessing

Bulk transcriptomic datasets were obtained from GEO using the GEOquery package (version 2.74.0) and processed using standard pipelines. GSE119600 (Illumina HumanHT-12 V4.0 beadChip; 47 control, 45 PSC) was normalized using normalizeBetweenArrays from limma (version 3.62.2), annotated with illuminaHumanv4. db (version 1.26.0), and log2-transformed. GSE177044 (RNA-seq; 78 control, 42 PSC) was filtered to retain genes expressed in ≥ 10% of samples, normalized using the median-of-ratios method implemented in DESeq2 (version 1.46.0), and transformed as log2 (normalized counts + 1). Gene symbols were harmonized across datasets, with AMICA1 renamed to JAML.

### Single-cell RNA-seq data processing

Stringent quality control procedures were applied during single-cell RNA-seq data processing. For each sample, cells were initially filtered to retain those with mitochondrial gene content < 40%, number of detected genes ≥ 200, and UMI count ≥ 500. Doublets were removed using scDblFinder (version 1.20.2) (Germain et al., 2046) with sample-specific expected doublet rates (3%–10% based on cell number). After merging individual samples using the merge function, an adaptive filtering step was performed based on the 99th percentile of the mitochondrial gene proportion per sample (upper limit 35%). Cells were further selected with UMI counts between 1,000 and 20,000, detected genes between 200 and 10,000, ribosomal gene proportion ≤ 25%, and hemoglobin gene proportion ≤ 5%. Data normalization and downstream analysis were carried out using Seurat (version 5.2.1) (Hao et al., 2013). The top 2,000 highly variable genes were identified, and data were scaled while regressing out the effects of UMI count and mitochondrial gene proportion. After dimensionality reduction by principal component analysis (PCA), batch effects across samples were corrected using Harmony (version 2.0.2) (Korsunsky et al., 2019) applied to the first 20 principal components. Finally, clustering was performed at a resolution of 0.5, and cell types were annotated based on differentially expressed genes and canonical markers.

### Transcriptome-wide association study

Transcriptome-wide association study (TWAS) integrates eQTL information to evaluate the association between genetically predicted gene expression and disease phenotypes (Mai et al., 2023). In this study, TWAS was performed using the FUSION software (Gusev et al., 2016). Briefly, gene expression weights were precomputed based on a reference panel of individuals with both genotype and expression data, and then applied to GWAS summary statistics to impute genetically predicted expression levels and test their association with PSC. The analysis used GTEx v8 liver tissue as the reference panel, PSC GWAS data (GCST004030), and a P-value threshold of < 0.01 for candidate gene selection. FUSION analysis employed the precomputed weight file for liver tissue (Liver.v8. normalized_expression.bed), with a minimum eQTL P-value threshold of 5 × 10^−8^, a 1 Mb window upstream and downstream of each gene, and the 1000 Genomes Project European population as the linkage disequilibrium (LD) reference panel.

### Functional enrichment analysis

To explore the potential biological significance of the candidate genes, we performed GO, KEGG, GSEA, and GSVA analyses. GO annotation was carried out from three aspects: biological process (BP), cellular component (CC), and molecular function (MF). KEGG provides a framework of molecular interaction and reaction networks. GSEA examines the enrichment trends of predefined gene sets based on differential expression rankings, while GSVA calculates sample-level gene set enrichment scores to quantify pathway activity. GO, KEGG, and GSEA were conducted using the clusterProfiler package (version 4.14.6) (Yu et al., 2012), and GSVA was performed using the GSVA package (version 2.0.7) (Hänzelmann et al., 2013). Gene sets were sourced from the MSigDB Hallmark collection (h.all.v2026.1. Hs.symbols.gmt) and the KEGG_MEDICUS collection (c2. cp.kegg_medicus.v2026.1. Hs.symbols.gmt).

### TWAS activity evaluation

A total of 219 PSC-related candidate genes were identified by TWAS at a threshold of P < 0.01. After intersecting with the single-cell expression matrix, 178 genes were retained for subsequent analysis. To evaluate TWAS activity at the single-cell level, we applied five gene set scoring methods implemented in irGSEA (version 3.3.4) (Fan et al., 2024): AUCell (version 1.28.0) (Aibar et al., 2017), UCell (version 2.10.1) (Andreatta and Carmona, 2021), AddModuleScore (Tirosh et al., 2016), singscore (version 1.26.0) (Foroutan et al., 2018), and ssGSEA (implemented in the GSVA framework) (Hänzelmann et al., 2013). For each scoring method, the resulting score matrix was Z-score standardized across all cells and then Min–Max normalized to [0,1]. The normalized scores from the five methods were summed per cell to obtain a composite score (Scoring). Based on quartiles of the composite score, cells were divided into low TWAS activity state (LTS, < 25th percentile), medium activity state (MTS, 25th–75th percentile), and high TWAS activity state (HTS, > 75th percentile).

### Subclustering of mononuclear phagocytes and functional analysis of LAM subsets

After extracting the monocyte/macrophage population from the global single-cell dataset, we repeated normalization, highly variable gene selection, scaling, and PCA, followed by UMAP visualization based on the first 15 principal components. Initial clustering was performed at a resolution of 0.4. Marker genes for each cluster were identified by differential expression analysis, and non-monocyte/macrophage clusters were removed based on known lineage markers (labeled as “Unknown”). The remaining cells were re-clustered (resolution 0.3) and annotated into five subsets based on differentially expressed genes and canonical markers: monocytes, inflammatory monocytes/macrophages (Inflam MoMF), lipid-associated macrophages (LAM Mac), antigen-presenting cell-like macrophages (APC-like Mac), and CD16^+^ monocytes (CD16^+^ Mono). Based on the quartiles of the TWAS composite score (Scoring), LAM macrophages were further divided into LTS, MTS, and HTS groups. Subsequently, GSVA was used to evaluate pathway activity: Hallmark gene sets were used for GSVA between HTS and LTS groups, while KEGG_MEDICUS gene sets were used for GSVA across HTS, MTS, and LTS groups.

### Identification of HTS signature genes

To identify key signature genes associated with the HTS state and to evaluate the contribution of TWAS candidate genes to the composite score, we performed feature selection using a combination of machine learning algorithms, including LASSO, random forest (RF), XGBoost, Boruta, gradient boosting machine (GBM), ABESS, and decision tree (DT). Feature selection and modeling were performed using seven algorithms. LASSO regression was applied for feature selection using the regularization framework proposed by Tibshirani (1996), with the penalty parameter *α* set to 1 and the tuning parameter *λ* determined via 5-fold cross-validation (lambda.min). Random Forest assessed feature importance based on the Gini coefficient (Feng et al., 2025), utilizing 1,000 trees (ntree) and a feature subset size of *p*​ (mtry). XGBoost was employed for feature extraction within a gradient boosting decision tree framework (Abu-Doleh and Al Fahoum, 2024), configured with 300 rounds, a maximum depth of 3, a learning rate (eta) of 0.05, and subsampling rates of 0.8 for both rows and columns. Boruta, an all-relevant feature selection method derived from Random Forest, was utilized to identify stable features (Hamidi et al., 2023) (100 maximum runs; α*α* = 0.01). Gradient Boosting Machine (GBM) enhanced classification by iteratively integrating weak learners (Hastie et al., 2009), with parameters set to 2,000 trees, an interaction depth of 3, a shrinkage of 0.01, a bag fraction of 0.7, and a minimum node size of 10. Additionally, ABESS was applied for adaptive optimal subset selection assuming a binomial family, and a decision tree model was constructed for supervised classification (Wani et al., 2025; Zhu et al., 2020). The seven algorithms were each applied to the binary classification task of HTS versus LTS in LAM macrophages, and the feature genes identified by each algorithm were intersected to obtain the core signature genes for subsequent analysis. To further evaluate the association between JAML and inflammatory pathway activity, we performed ssGSEA to compare TNFα/NF-κB and IL-6/JAK/STAT3 pathway activity between JAML+ and JAML- PSC LAMs. The proportion of JAML-high versus JAML-low LAMs was compared between PSC and PBC using Fisher’s exact test. A module score based on the 7 core HTS signature genes was calculated using the AddModuleScore function. In bulk RNA-seq datasets GSE119600 and GSE177044, Spearman correlation was used to assess the association between JAML expression and pathway ssGSEA scores in PSC samples.

### Pseudotime analysis

Monocle2 (monocle package version 2.34.0) (Trapnell et al., 2014) was used to construct the differentiation trajectory of mononuclear phagocytes. Using the raw count matrix as input, highly variable genes were selected for ordering. Dimensionality reduction and cell ordering were performed via the DDRTree method, with monocytes set as the root state of the trajectory. The trajectory was colored by cell type and pseudotime, respectively. The expression level of JAML in LAM macrophages was extracted and analyzed along pseudotime, and comparisons were made between TWAS state groups.

### Cell–cell communication analysis

To dissect ligand–receptor interactions within the PSC microenvironment, we performed cell–cell communication network inference on the annotated single-cell data using CellChat (version 1.6.1) (Jin et al., 2021). Based on the major cell types, LAM macrophages were further subdivided into LAM_HTS, LAM_MTS, and LAM_LTS, and the “Secreted Signaling” subset of the human CellChatDB was used as the reference database. The number and strength of communications among different cell types were compared, and the outgoing/incoming contributions of each cell group in the signaling network were evaluated. Communication patterns of the top 15 signaling pathways were compared. Furthermore, differences between LAM_HTS and LAM_LTS as signal senders or receivers were assessed, and significantly enriched ligand–receptor pairs were identified.

### Single-gene validation and immune infiltration functional analysis of JAML

Based on the seven core genes in the training set GSE119600, a random forest model was constructed to evaluate their contribution to PSC discrimination, and feature importance was measured by the MeanDecreaseGini index. The gene with the highest importance, JAML, was selected for subsequent validation (Cantor et al., 2024). The Wilcoxon rank-sum test was then used to compare JAML expression between normal and PSC groups in both GSE119600 and the independent validation set GSE177044, and the area under the ROC curve (AUC) was calculated. In the training set, PSC samples were divided into high- and low-expression groups based on the median JAML expression level. Gene set enrichment analysis (GSEA) was performed on the Hallmark gene set collection (h.all.v2026.1. Hs.symbols.gmt) (Candia and Ferrucci, 2024; Liberzon et al., 2015), and the top five pathways with the highest normalized enrichment scores were displayed. Immune cell abundance was evaluated using ssGSEA (implemented in the GSVA framework) (Hänzelmann et al., 2013), and enrichment scores for each immune cell type were calculated via GSVA to compare differences between JAML high- and low-expression groups. Additionally, Spearman rank correlation analysis was used to assess the correlation between JAML expression and immune cell abundance.

### Spatial transcriptomics data analysis

Spatial transcriptomics data were downloaded from GEO (GSE240429: healthy control; GSE245620: PSC), each containing four consecutive tissue sections (10x Genomics Visium FFPE). The expression matrix and spatial coordinates were read using the Seurat package. Only tissue-containing spots (in_tissue = 1) were retained, and each spot was filtered to have at least 200 genes and 500 UMIs. Data were normalized using SCTransform. After PCA dimensionality reduction, the first 30 principal components were used for UMAP visualization and graph-based clustering (resolution = 0.5). Module scores for two gene sets were calculated using AddModuleScore: the fibrosis gene set (COL1A1, COL3A1, ACTA2, TGFB1, TIMP1) (Staten et al., 2012) and the LAM (lipid-associated macrophage) gene set (TREM2, GPNMB, APOC1, SPP1, ACP5) (Fabre et al., 2023; Xu et al., 2025). Spatial distributions of JAML expression and module scores were visualized using SpatialFeaturePlot (low-res scaling, point size factor = 2, with a fixed upper color limit of 0.8 for JAML expression). Coordinates and scores for each spot were extracted, and Spearman rank correlation was used to assess the associations of JAML with LAM score, JAML with fibrosis score, and LAM score with fibrosis score. JAML positivity was defined as expression > 0, and the Wilcoxon rank-sum test was used to compare fibrosis scores between JAML-positive and JAML-negative groups. Each section was analyzed independently, and results are presented as ranges of correlation coefficients. Two-sided P < 0.05 was considered statistically significant.

### Single-cell simulation of gene knockout analysis (scTenifoldKnk)

To assess the functional importance of JAML in LAM macrophages, we performed virtual gene knockout using the scTenifoldKnk package (version 1.0.3) (Osorio et al., 2022). First, LAM macrophages expressing JAML (count > 0) were selected. Genes expressed in fewer than 20% of these cells or with total counts < 50 were filtered out. The filtered count matrix was then used as input for the knockout analysis. The scTenifoldKnk function was run with the following parameters: gKO = “JAML”, qc = TRUE, qc_mtThreshold = 0.2, qc_minLSize = 800, nc_nCells = min (400, nCells), nc_nNet = 10, td_K = 5, and nCores = 2. A gene was considered significantly perturbed if its Benjamini–Hochberg-adjusted P-value was < 0.05. The perturbation magnitude was defined as the log_2_ fold change of the gene’s regulatory influence. Finally, Gene Ontology (GO) enrichment analysis of the significantly perturbed genes was performed using the clusterProfiler package, with an adjusted P < 0.05 as the significance threshold.

### *In vitro* macrophage stimulation and western blot validation

Human monocytic THP-1 cells were differentiated into macrophage-like cells using 100 nM phorbol 12-myristate 13-acetate (PMA) for 24 h, followed by a 24-h rest in fresh RPMI 1640 medium supplemented with 10% fetal bovine serum. Cells were then stimulated with 100 ng/mL lipopolysaccharide (LPS) plus 20 ng/mL recombinant human IL-6 for 24 h to model inflammatory activation. Where indicated, cells were pre-treated with 10 μM BAY 11–7,082 (an NF-κB inhibitor) for 1 h before cytokine stimulation. For JAML knockdown, cells were transfected with 50 nM JAML-targeting siRNA or negative-control siRNA using Lipofectamine RNAiMAX according to the manufacturer’s instructions, and stimulated 48 h after transfection. Total protein was extracted using RIPA lysis buffer, separated by SDS-PAGE, and transferred to PVDF membranes. Immunoblotting was performed with primary antibodies against JAML, NF-κB p65, phospho-NF-κB p65 (Ser536), and PD-L1/CD274 (Proteintech, Wuhan, China), followed by HRP-conjugated secondary antibodies and chemiluminescent detection. IL-6 concentrations in culture supernatants were measured by ELISA. All key comparison groups were run on the same membrane to ensure direct comparability.

### Transwell co-culture model for mechanistic discussion

For mechanistic discussion, we outline a Transwell co-culture system in which LPS/IL-6-activated macrophages are seeded in the upper insert and human cholangiocyte H69 cells in the lower chamber. Conditioned-medium transfer and NF-κB/IL-6 pathway inhibition experiments are described as follow-up approaches to test whether JAML-positive macrophages drive cholangiocyte PD-L1 expression through IL-6/NF-κB signaling.

### Statistical analysis

All statistical analyses were performed in R (version 4.4.1). Comparisons between two independent groups were conducted using the Wilcoxon rank-sum test. Correlations were assessed using Spearman’s rank correlation coefficient. All tests were two-sided, and P < 0.05 was considered statistically significant unless otherwise specified. For multiple testing, the false discovery rate was controlled using the Benjamini–Hochberg method.

## Results

### TWAS analyses identified PSC-related genes

**FIGURE 1:**
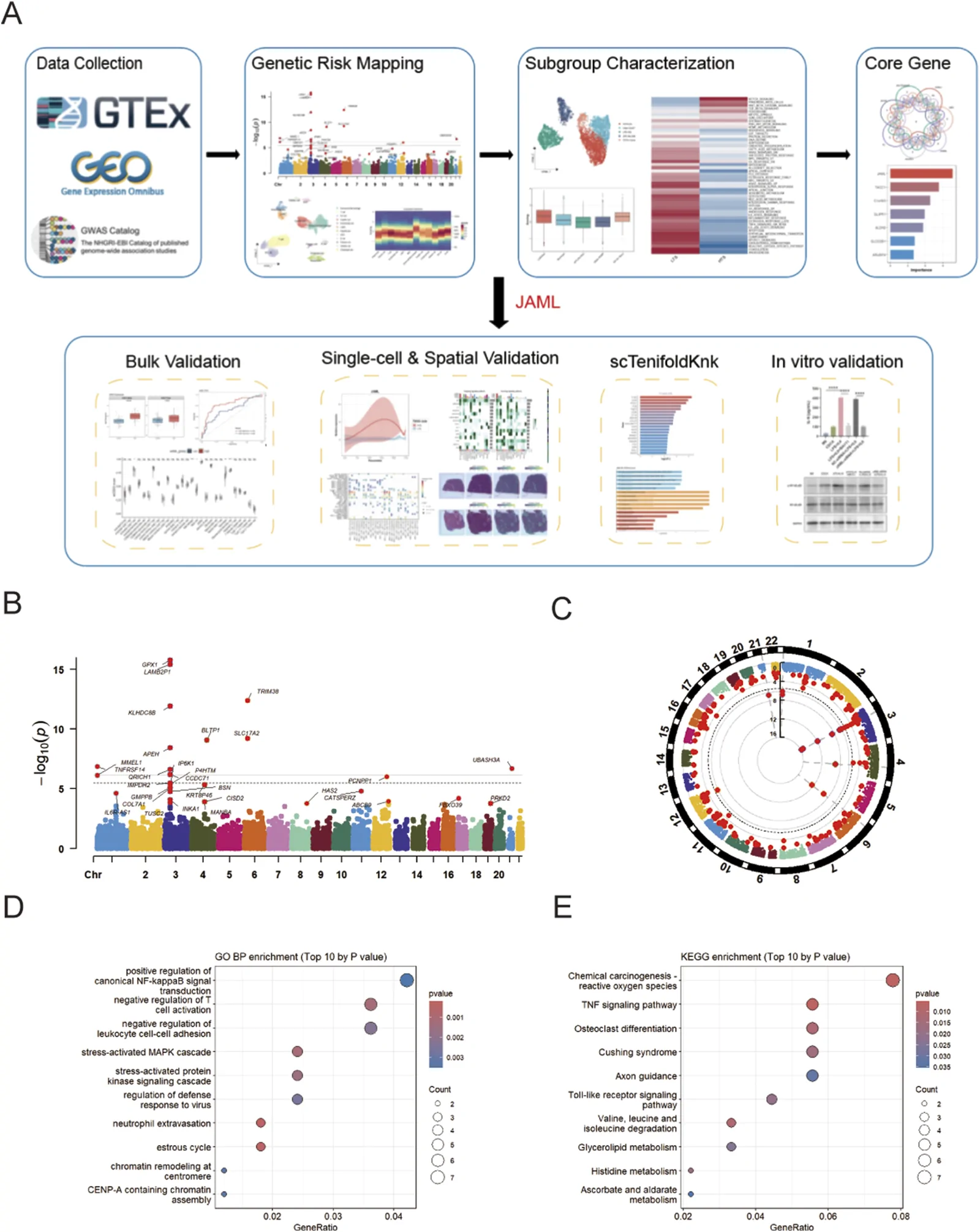
The identification of PSC-associated genes by TWAS. **(A)** Flowchart of the overall study design. **(B)** Rect plot of TWAS results for primary sclerosing cholangitis (PSC). **(C)** Circular Manhattan plot of TWAS for PSC. **(D)** GO enrichment analysis of the TWAS-prioritized genes. **(E)** KEGG enrichment analysis of the TWAS-prioritized genes.

The overall analytical workflow of this study is illustrated in Figure 1A, consisting of five main stages: data collection (GTEx, GEO, GWAS Catalog), genetic risk mapping (TWAS), subgroup characterization (single-cell transcriptomics), core gene identification (machine learning), and validation with functional inference (bulk validation, single-cell/spatial validation, and virtual knockout). In the TWAS analysis, 219 genes with P < 0.01 were identified (Figures 1B,C; Additional file 1). To explore the potential biological relevance of these genes, we performed GO and KEGG enrichment analyses. GO biological process terms were enriched for immune- and inflammation-related processes, including positive regulation of canonical NF-κB signaling, negative regulation of T-cell activation, and neutrophil extravasation (Figure 1D). KEGG analysis showed enrichment of pathways such as TNF signaling, Toll-like receptor signaling, and chemical carcinogenesis–reactive oxygen species (Figure 1E). Together, these results suggest that TWAS-prioritized genes may be involved in immune regulation, inflammatory activation, and microenvironment remodeling in PSC.

### scRNA-seq analysis reveals PSC cellular atlas and TWAS activity heterogeneity

**FIGURE 2:**
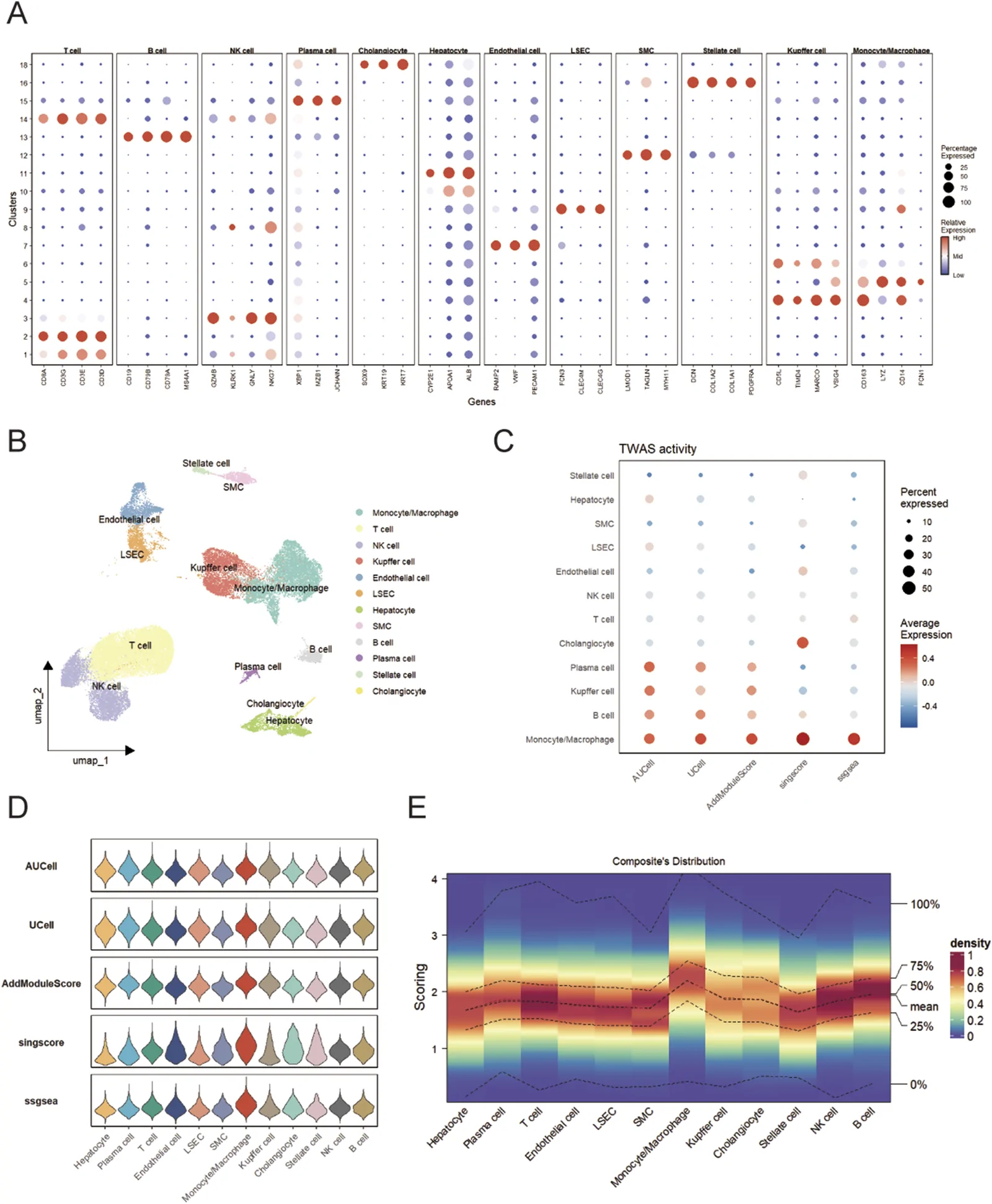
Single-cell atlas of PSC liver and TWAS activity scoring across cell types. **(A)** Dot plot showing representative marker genes for each cell type. **(B)** UMAP plot showing the 12 major cell types annotated in PSC liver samples. **(C)** Dot plots showing the TWAS activity scores measured by five algorithms (AUCell, UCell, AddModuleScore, singscore, and ssGSEA). **(D)** Violin plots showing the distribution of TWAS activity scores measured by the five algorithms. **(E)** Heatmap showing the distribution of the integrated composite score (Scoring) across 12 cell types.

A total of 40,002 cells from eight PSC liver samples were retained after quality control for downstream analysis. After Harmony integration, cells from different samples showed comparable distributions in the low-dimensional embedding, supporting effective batch correction. Using the Seurat pipeline, we identified 18 clusters and annotated them into 12 major cell types based on canonical markers, including T cells, B cells, NK cells, plasma cells, cholangiocytes, hepatocytes, endothelial cells, liver sinusoidal endothelial cells (LSECs), smooth muscle cells, stellate cells, Kupffer cells, and monocytes/macrophages. Figure 2A shows canonical marker expression across annotated cell types, and Figure 2B displays the UMAP embedding. To quantify TWAS activity at single-cell resolution, we applied five scoring methods (AUCell, UCell, AddModuleScore, singscore, and ssGSEA) and an integrated composite score (Scoring). Monocytes/macrophages showed the highest TWAS activity among major cell types (Figures 2C–E), suggesting that PSC genetic risk-related transcriptional activity is preferentially enriched in the monocyte/macrophage lineage. To assess the specificity of the PSC-associated cellular landscape, we compared the cellular composition across CONTROL, PBC, and PSC samples from the same dataset. Relative to CONTROL, PBC and PSC samples exhibited markedly higher proportions of immune cells and reduced proportions of non-immune cells, including hepatocytes and cholangiocytes (Additional file 2: Supplementary Figure S1B), consistent with the pathological features of immune cell infiltration in PBC/PSC and reflecting the advanced fibrotic stage of the PSC samples analyzed. JAML expression was low in CONTROL, elevated in PBC, and highest in PSC (Additional file 2: Supplementary Figure S1D, left), suggesting that JAML upregulation is associated with cholestatic liver disease rather than a general feature of healthy liver macrophages. Notably, JAML expression in PBC was predominantly restricted to myeloid cells, whereas in PSC it was detected in both myeloid and T-cell compartments (Additional file 2: Supplementary Figure S1D, right).

### Sub-clustering of the monocyte–macrophage lineage and identification of LAM macrophages

**FIGURE 3:**
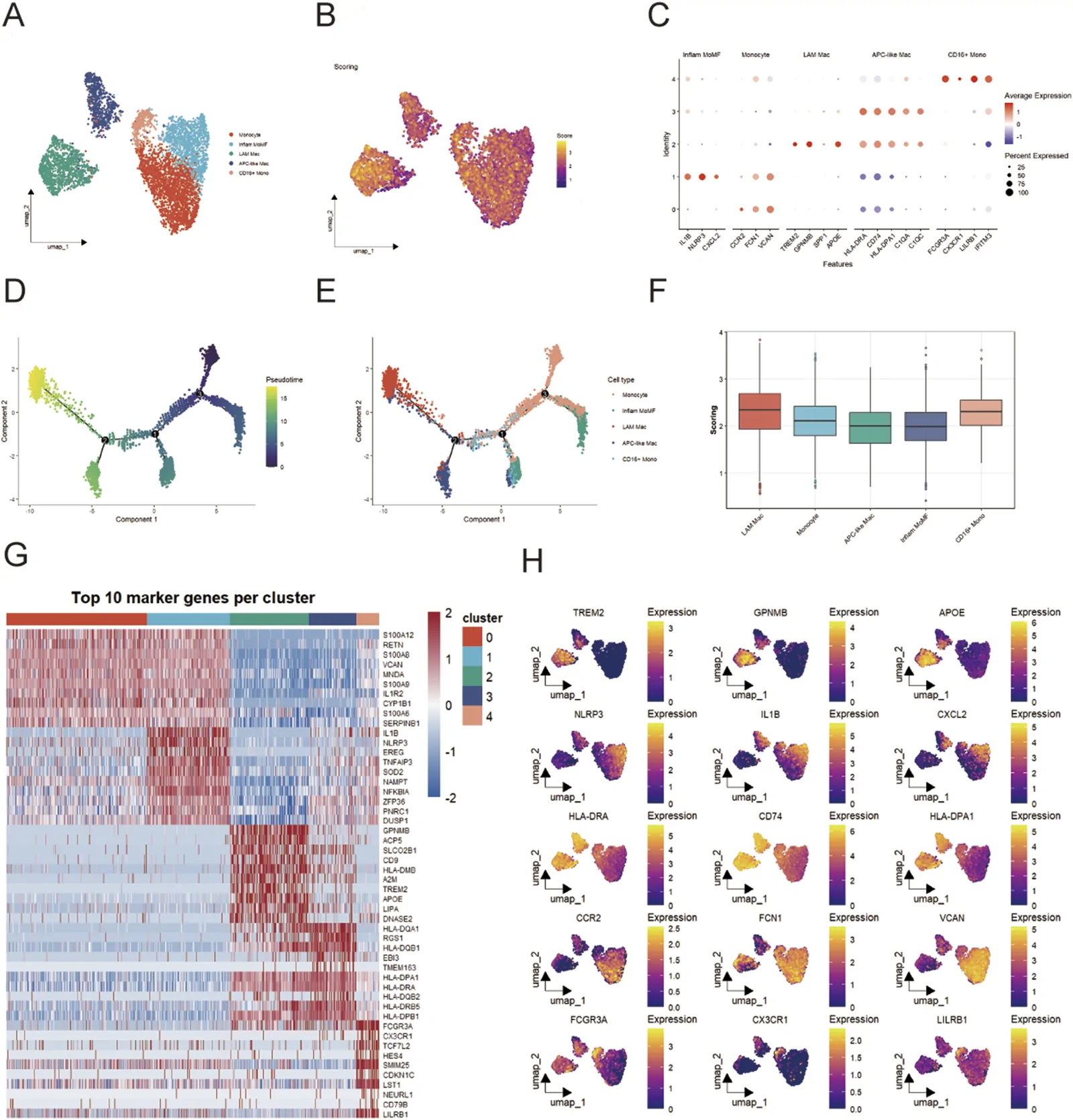
Sub-clustering of the monocyte–macrophage lineage and identification of LAM macrophages with high TWAS activity. **(A)** UMAP plot showing five subsets of mononuclear phagocytes. **(B)** UMAP plot with projection of the TWAS composite score, revealing higher activity in the LAM macrophage region. **(C)** Heatmap showing the expression profiles of marker genes for each subset. **(D)** Pseudotime trajectory analysis of mononuclear phagocytes with monocytes set as the root, showing the trajectory colored by pseudotime. **(E)** Pseudotime trajectory analysis colored by cell type. **(F)** Boxplot comparing TWAS activity across the five subsets, demonstrating significantly higher activity in LAM macrophages. **(G)** Subset-specific marker gene heatmap validating the accuracy of cell type annotation. **(H)** UMAP plot showing representative marker genes for each cell type.

To further refine the mononuclear phagocyte subset with the highest TWAS activity, we performed re-clustering and annotation of this lineage, which identified five subsets: monocytes, inflammatory monocytes/macrophages (Inflam MoMF), lipid-associated macrophages (LAM Mac), antigen-presenting cell-like macrophages (APC-like Mac), and CD16^+^ monocytes (CD16^+^ Mono) (Figure 3A). Projection of the TWAS composite score onto the UMAP plot revealed that the LAM macrophage region exhibited higher activity levels (Figure 3B). The expression profiles of marker genes for each subset were consistent with previous reports: LAM macrophages highly expressed TREM2, GPNMB, SPP1, and APOE (Ramachandran et al., 2019; Xiong et al., 2019); monocytes expressed CCR2, FCN1, and VCAN (Tsou et al., 2007; Villani et al., 2017); inflammatory monocytes/macrophages expressed IL1B, NLRP3, and CXCL2 (Martinon et al., 2002; He et al., 2016; De Filippo et al., 2013); CD16^+^ monocytes expressed FCGR3A, CX3CR1, LILRB1, and IFITM3 (Villani et al., 2017; Auffray et al., 2007; Cros et al., 2010); and APC-like macrophages expressed HLA-DRA, CD74, HLA-DPA1, C1QA, and C1QC (Roche and Furuta, 2015; Han et al., 2020) (Figure 3C). The subset-specific marker gene heatmap and FeaturePlots further validated the accuracy of this annotation (Figures 3G,H). Pseudotime trajectory analysis, with monocytes set as the differentiation root, showed that monocytes and inflammatory monocytes/macrophages were positioned at the early stage of differentiation, whereas LAM macrophages were localized to the late stage (Figures 3D,E). Quantitative comparison demonstrated that TWAS activity in LAM macrophages was significantly higher than that in the other four subsets (Figure 3F). Collectively, these results identify LAM macrophages as a terminally differentiated subset with the highest TWAS activity in PSC. To assess whether the myeloid subsets identified in PSC were also present in other disease conditions, we performed an additional re-clustering analysis integrating CONTROL, PBC, and PSC samples. Under conditions where myeloid cells were present, the five major subsets observed in the PSC-only analysis (monocytes, Inflam MoMF, LAM Mac, APC-like Mac, and CD16^+^ monocytes) were consistently identified in both PBC and PSC (Additional file 2: Supplementary Figures S1E,F), indicating that the partitioning of myeloid cells into these subsets is reproducible across different cholestatic liver diseases. Notably, in CONTROL samples, the low overall abundance of myeloid cells resulted in correspondingly fewer cells in these subsets, consistent with the physiological characteristic of low immune cell infiltration in normal liver.

### LAM macrophage TWAS activity stratification and functional heterogeneity

**FIGURE 4:**
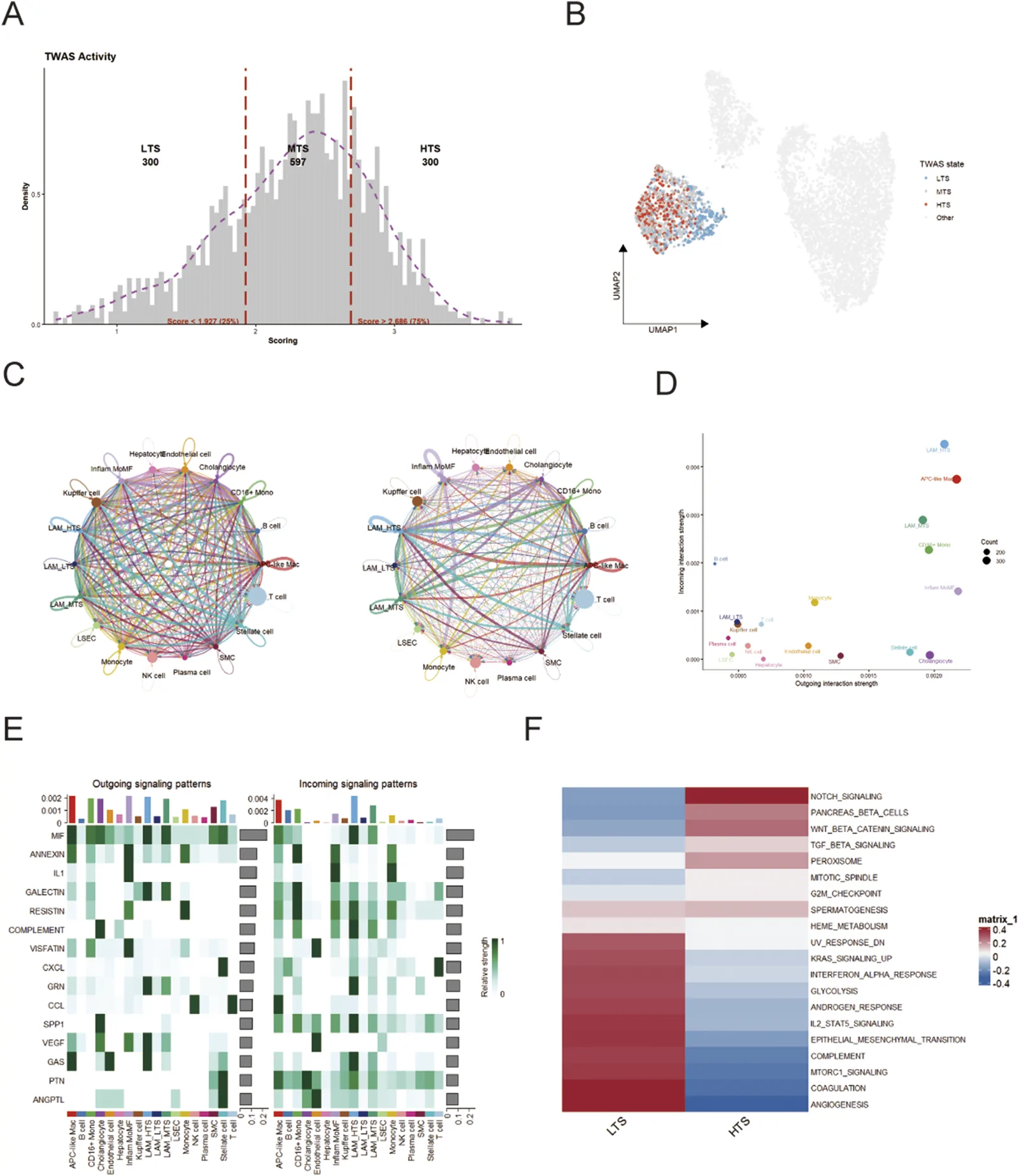
Stratification of LAM macrophages by TWAS activity and functional heterogeneity. **(A)** Distribution of TWAS activity in LAM macrophages, showing stratification into low (LTS), medium (MTS), and high (HTS) activity states based on composite score quartiles. **(B)** UMAP projection showing spatial separation between HTS and LTS cells. **(C)** Cell–cell communication network showing the number and strength of interactions between LAM_HTS and LAM_LTS with other cell types. **(D)** Scatter plot of the inferred roles of cell types based on incoming and outgoing interaction strength. **(E)** Signaling role analysis of the cell–cell communication network, highlighting pathways with stronger outgoing or incoming signals in LAM_HTS cells. **(F)** Hallmark gene set GSVA comparing HTS and LTS groups, showing pathway enrichment differences between the two states. More abundant ligand–receptor interactions in the MIF pathway are shown in Additional file 3: Supplementary Figure S2A. KEGG_MEDICUS-based GSVA across HTS, MTS, and LTS groups is provided in Additional file 3: Supplementary Figure S2B.

To dissect the heterogeneity of TWAS activity within LAM macrophages, we stratified them into LTS, MTS, and HTS groups based on the quartiles of the composite Scoring metric (Figure 4A). UMAP projection revealed a spatial separation between HTS and LTS cells (Figure 4B). CellChat analysis demonstrated that, compared with LAM_LTS cells, LAM_HTS cells exhibited stronger outgoing and incoming signaling contributions (Figure 4C). They also showed higher overall communication number and strength (Figure 4D). Among the top 15 signaling pathways, LAM_HTS cells showed stronger outgoing signaling in pathways such as MIF, GALECTIN, and VEGF, and stronger incoming signaling in MIF, COMPLEMENT, and SPP1 (Figure 4E); the more abundant ligand–receptor interactions and higher signaling intensity in the MIF pathway are shown in Additional file 3: Supplementary Figure S2A. At the functional level, Hallmark gene set GSVA revealed that the LTS group was mainly enriched in pathways such as angiogenesis, coagulation, reactive oxygen species, cholesterol homeostasis, and mTORC1 signaling, whereas the HTS group was significantly enriched in NOTCH, WNT/β-catenin, TGF-β signaling, and peroxisome pathways (Figure 4F). KEGG_MEDICUS-based GSVA further indicated that the HTS group exhibited relative upregulation of NLRP3 inflammasome regulation, calcium transport, CASR/PTH signaling, NOTCH-MESP2 signaling, peroxisomal β-oxidation, and TLR2/4-MAPK signaling pathways (Additional file 3: Supplementary Figure S2B). Collectively, these results suggest that the HTS state may represent a functional phenotype of LAM macrophages with enhanced inflammatory, fibrosis-related, and cell-communication activities.

### Identification of HTS signature genes by multiple machine learning algorithms

**FIGURE 5:**
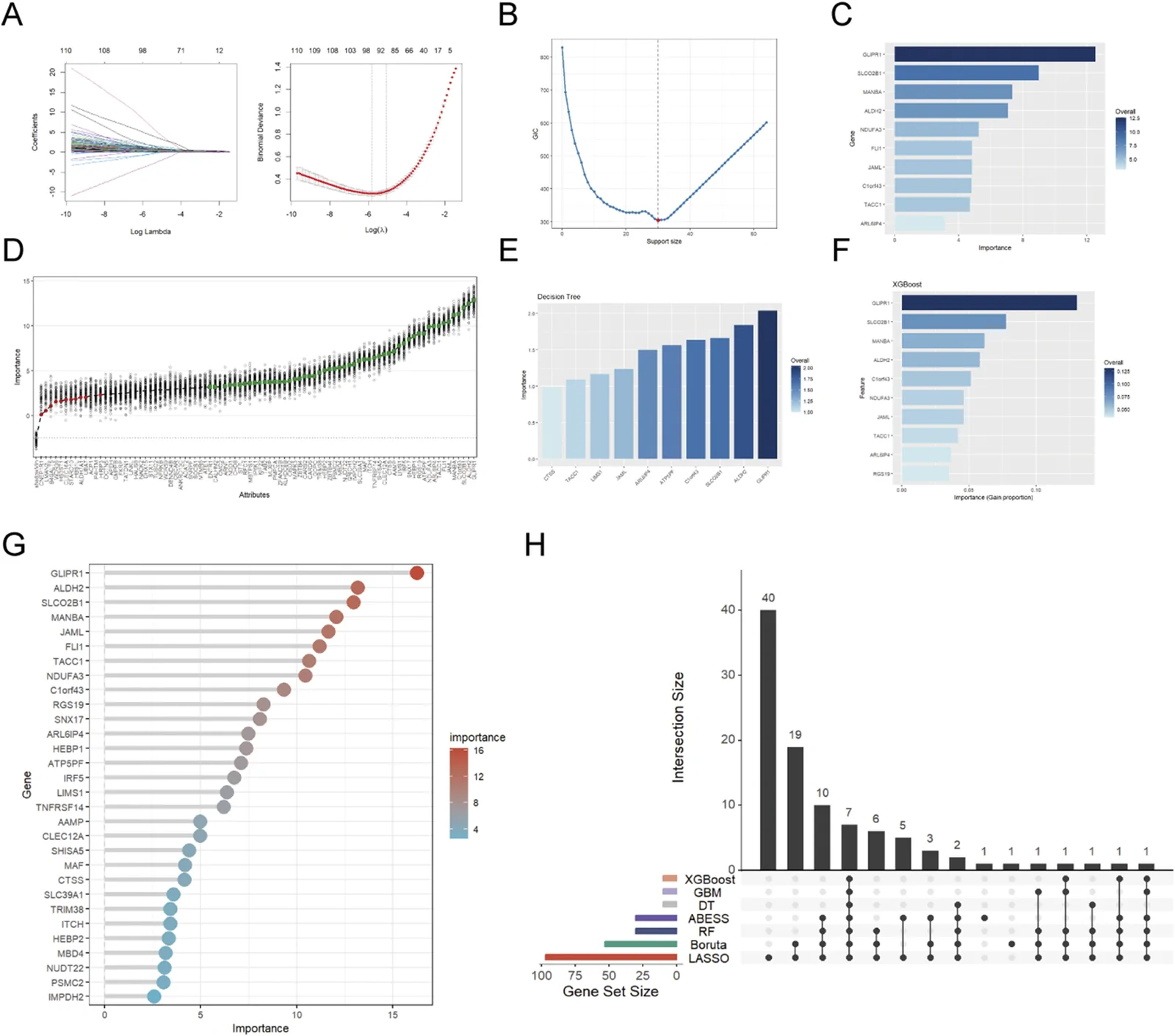
Identification of HTS signature genes by multiple machine learning algorithms. **(A)** LASSO coefficient profiles of the candidate feature genes; the optimal lambda was determined when the partial likelihood deviance reached the minimum value. **(B)** The ABESS algorithm selected feature genes based on the GIC criterion. **(C)** GBM algorithm displayed the top feature genes ranked by importance. **(D)** Boruta algorithm confirmed relevant feature genes. **(E)** Decision tree model selected the top feature genes. **(F)** XGBoost algorithm extracted the top feature genes based on Gain. **(G)** Random forest model obtained the top feature genes ranked by the Gini coefficient; the distribution of feature importance is visualized as a lollipop plot. **(H)** UpSet plot showing the intersection of feature genes identified by the seven algorithms, yielding the core signature genes.

To identify key signature genes associated with the HTS state, we performed a binary feature selection using HTS as the positive class and LTS as the negative class. LASSO regression identified 97 genes with non-zero coefficients (Figure 5A). The ABESS algorithm selected 30 feature genes based on the GIC criterion (Figure 5B). GBM extracted the top 10 genes ranked by feature importance (Figure 5C). Boruta further confirmed 53 relevant feature genes (Figure 5D). The decision tree model selected the top 10 feature genes (Figure 5E). XGBoost extracted the top 10 genes based on Gain (Figure 5F). The random forest model obtained the top 30 genes ranked by the Gini coefficient, with the distribution of feature importance visualized as a lollipop plot (Figure 5G). Finally, the intersection of the selection results from the seven algorithms was visualized by an UpSet plot, yielding seven core signature genes: JAML, TACC1, C1orf43, GLIPR1, ALDH2, SLCO2B1, and ARHGIP4 (Figure 5H). The complete gene lists selected by each algorithm are provided in Additional file 4. To validate the HTS signature at the population level, we computed a module score based on the 7 core HTS signature genes identified by multi-algorithm intersection (JAML, TACC1, C1orf43, GLIPR1, ALDH2, SLCO2B1, and ARHGIP4). LAM macrophages from PSC exhibited significantly higher module scores compared with those from PBC (Additional file 5: Supplementary Figure S3A), indicating enrichment of the HTS signature in PSC LAMs.

### Single-gene validation and functional analysis of JAML

**FIGURE 6:**
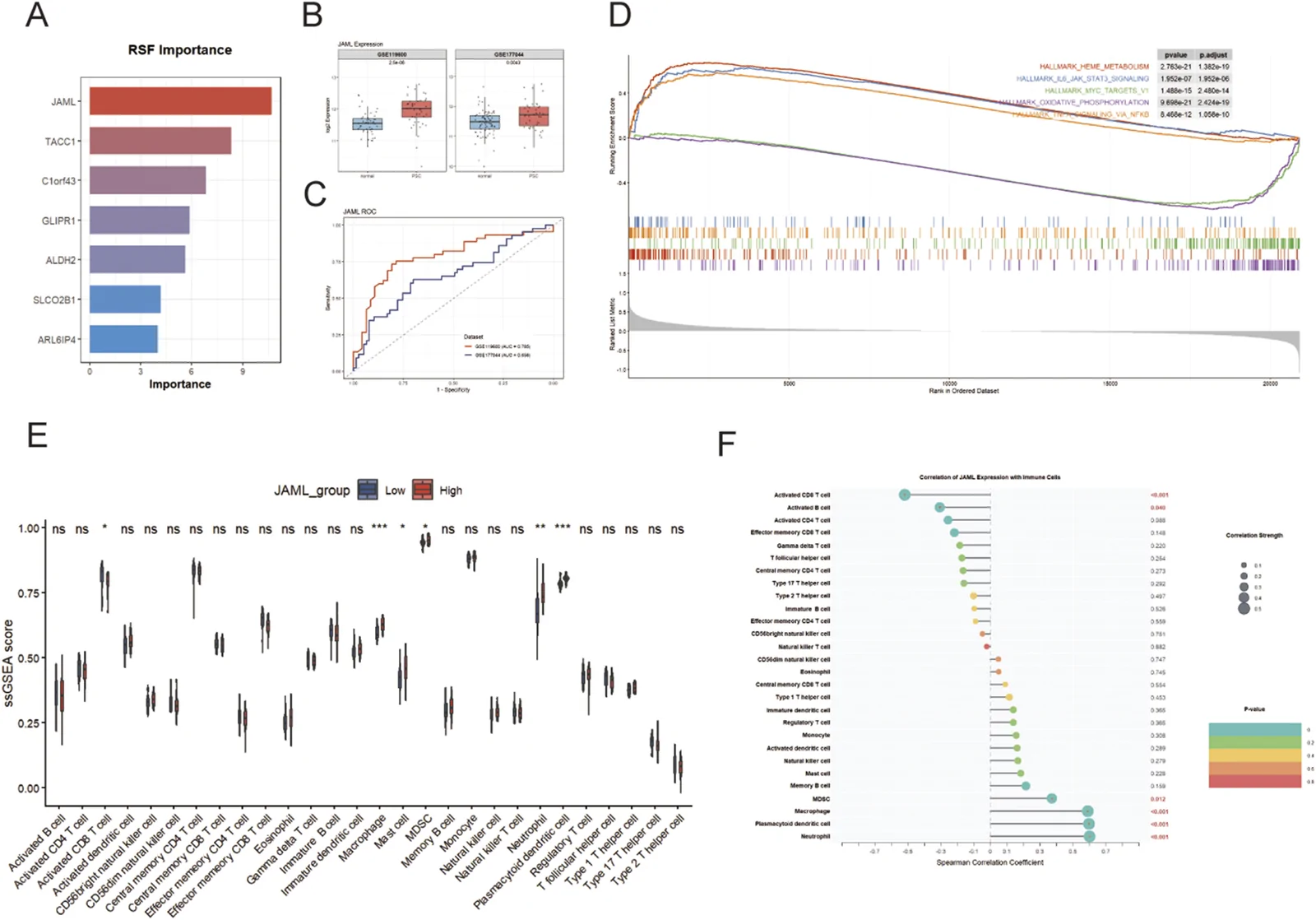
Single-gene validation and functional analysis of JAML. **(A)** Random forest feature importance plot showing JAML as the top-ranked gene. **(B)** Boxplots showing JAML expression upregulated in PSC compared to controls. **(C)** ROC curves demonstrating the discriminative ability of JAML for PSC. **(D)** GSEA enrichment plots comparing JAML-high and JAML-low expression groups. **(E)** Immune infiltration analysis showing differences in immune cell abundance between JAML-high and JAML-low groups. **(F)** Spearman correlation analysis showing the association of JAML expression with immune cell infiltration.

After constructing a random forest model based on the seven core genes in the training set GSE119600, JAML showed the highest importance score (MeanDecreaseGini) among all features (Figure 6A). JAML expression was significantly upregulated in the PSC group compared with controls in both the training set and the independent validation set (GSE177044) (Figure 6B), with area under the ROC curve (AUC) values of 0.785 and 0.658, respectively (Figure 6C). Further functional analysis revealed that the JAML-high expression group was significantly enriched in inflammation-related pathways, including IL6_JAK_STAT3_SIGNALING and TNFA_SIGNALING_VIA_NFKB, as well as HEME_METABOLISM, whereas the JAML-low expression group was enriched in MYC_TARGETS_V1 and OXIDATIVE_PHOSPHORYLATION pathways (Figure 6D). Immune infiltration analysis showed that the JAML-high group exhibited a significantly higher abundance of macrophages and a significantly lower abundance of CD8^+^ T cells (Figure 6E). Spearman correlation analysis further demonstrated that JAML expression was positively correlated with macrophage infiltration and negatively correlated with activated CD8^+^ T cell infiltration (Figure 6F). To further validate the association between JAML and inflammatory pathway activation at the single-cell level, we stratified PSC LAM macrophages into JAML+ and JAML-groups based on JAML expression. JAML + LAMs exhibited significantly higher TNFα/NF-κB and IL-6/JAK/STAT3 pathway activity compared with JAML- LAMs (Additional file 5: Supplementary Figure S3B), consistent with the correlations observed in bulk RNA-seq data (Additional file 5: Supplementary Figure S3D), supporting the association between JAML and inflammatory pathway activation at both single-cell and bulk levels. Additionally, the proportion of JAML-high LAMs was significantly higher in PSC compared with PBC (Additional file 5: Supplementary Figure S3C), further supporting the enrichment of the JAML + LAM state in PSC.

### Dynamic expression of JAML and its role in cell–cell communication

**FIGURE 7:**
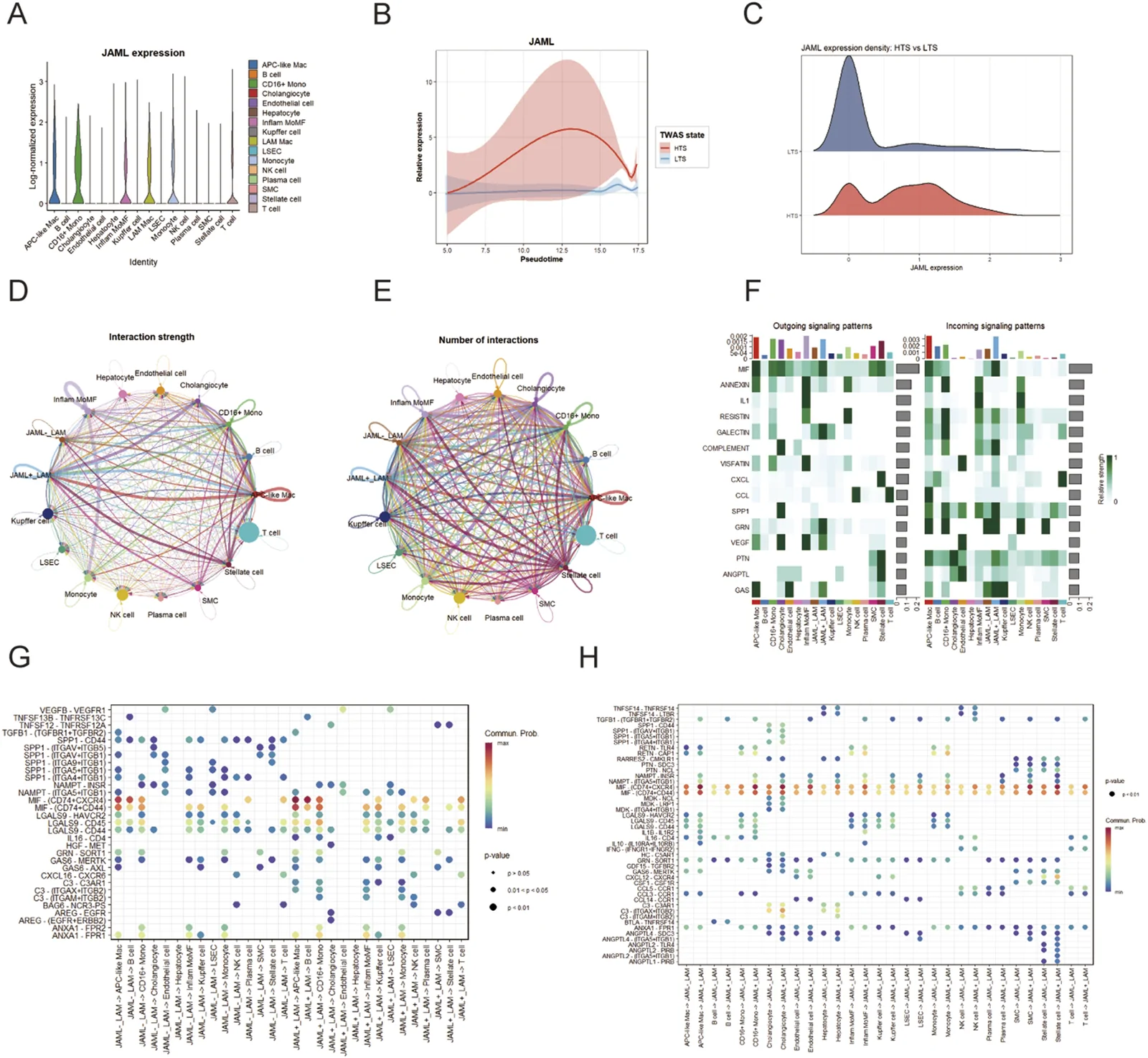
Dynamic expression of JAML and its role in cell–cell communication. **(A)** Dot plot showing JAML expression across global cell types. **(B)** Pseudotime trajectory showing dynamic changes of JAML expression during LAM differentiation. **(C)** Density plot comparing JAML expression between HTS and LTS cells. **(D,E)** Comparison of overall communication strength **(D)** and number **(E)** between JAML+ and JAML- LAM cells. **(F)** Outgoing and incoming signaling contribution of JAML + versus JAML- LAM cells. **(G, H)** Ligand-receptor bubble diagram of different cell types, with JAML^+^ LAM cells as senders **(G)** and receivers **(H)**.

At the global cell type level, JAML was predominantly expressed in the monocyte/macrophage lineage and T cells (Figure 7A). Pseudotime trajectory analysis revealed that, during LAM differentiation, JAML expression in HTS cells exhibited a dynamic pattern of initial increase followed by a decrease, whereas JAML expression in LTS cells remained consistently low with little fluctuation (Figure 7B). A comparison of JAML expression density between HTS and LTS cells is shown in Figure 7C. To evaluate the association between JAML and cell–cell communication, we stratified LAM macrophages into JAML + LAM and JAML- LAM groups based on JAML expression and performed CellChat analysis. The results showed that JAML + LAM cells exhibited higher overall communication strength and number compared with JAML- LAM cells (Figures 7D,E), as well as stronger outgoing and incoming signaling contributions in the signaling network (Figure 7F). At the pathway level, JAML + LAM cells showed stronger outgoing signaling in pathways such as MIF, GALECTIN, and VEGF, and stronger incoming signaling in MIF, COMPLEMENT, and SPP1 (Figure 7F). Further ligand–receptor interaction analysis demonstrated that JAML + LAM cells exhibited enhanced interaction strength with multiple cell types both as senders and receivers (Figures 7G,H; Figure 7G shows the sender perspective, and Figure 7H shows the receiver perspective). Collectively, these results suggest that JAML-high LAM macrophages possess enhanced cell–cell communication capabilities and may participate in the immune microenvironment regulation of PSC through multiple inflammation- and fibrosis-related signaling pathways.

### Virtual knockout of JAML using scTenifoldKnk perturbs the transcriptional network of LAM macrophages

**FIGURE 8:**
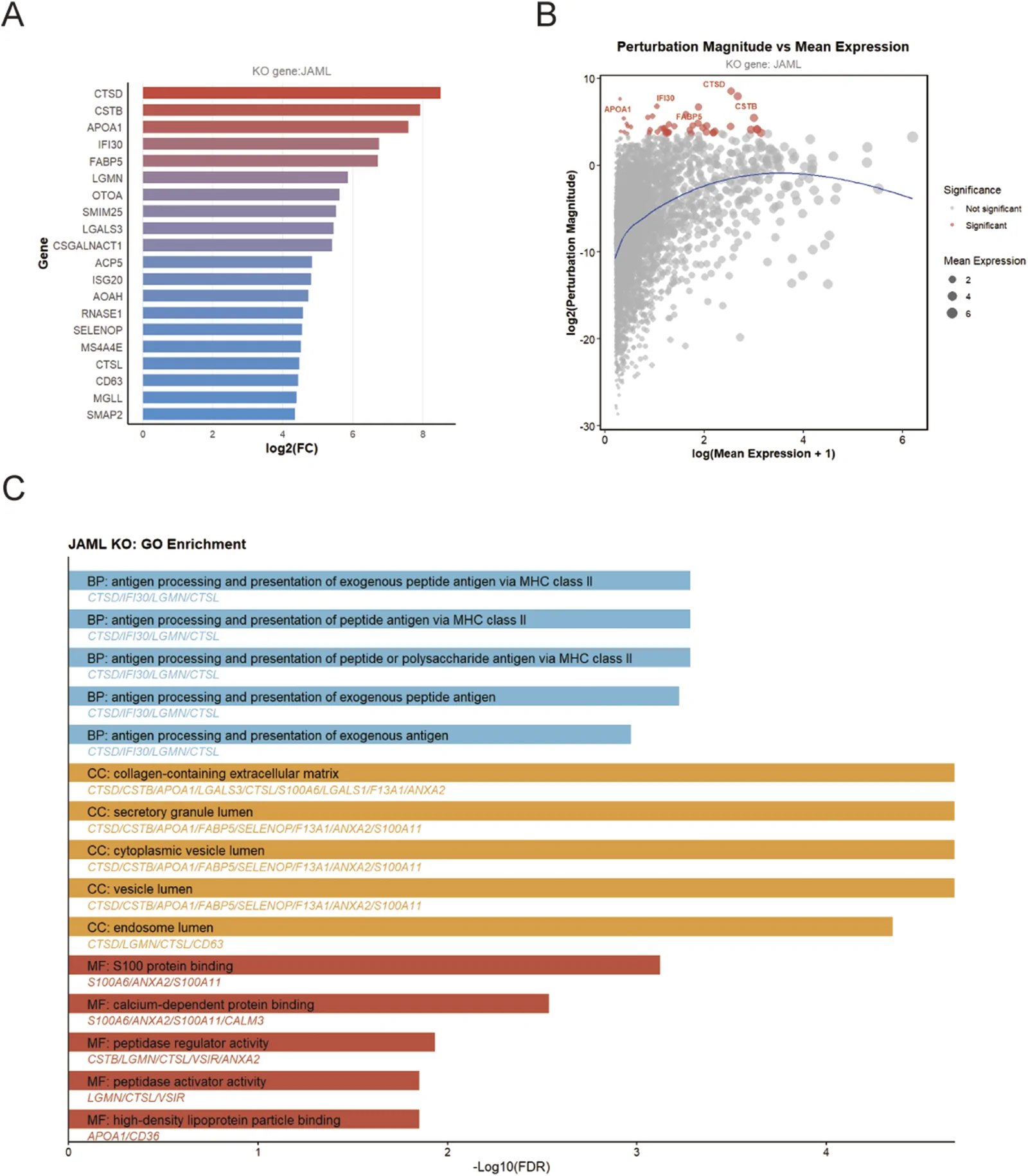
Virtual knockout of JAML perturbs the transcriptional network of LAM macrophages. **(A)** Bar plot showing genes ranked by perturbation magnitude. **(B)** Scatter plot showing the relationship between perturbation magnitude and mean expression. **(C)** GO enrichment analysis of perturbed genes.

After performing virtual knockout of JAML + LAM macrophages using scTenifoldKnk, a total of 45 significantly perturbed genes were identified with an adjusted P < 0.05 threshold. The top 20 genes ranked by log2 perturbation magnitude are shown as a horizontal bar plot (Figure 8A), and the relationship between perturbation magnitude and mean expression is presented in Figure 8B. GO enrichment analysis revealed that the perturbed genes were mainly enriched in MHC class II antigen processing and presentation (biological processes); collagen-containing extracellular matrix, vesicle lumen, and endosome lumen (cellular components); and peptidase regulator/activator activities, S100 protein binding, and calcium-dependent protein binding (molecular functions) (Figure 8C). The complete results of perturbation magnitude and significance for all genes are provided in Additional file 7, and the full GO enrichment results of perturbed genes are provided in Additional file 8. Collectively, these results suggest that JAML deficiency may perturb transcriptional networks associated with antigen presentation, extracellular matrix remodeling, and lysosomal/vesicle functions in LAM macrophages, supporting a potential role of JAML in maintaining the activated phenotype of these cells.

#### Spatial and *in vitro* validation of the JAML/NF-κB/PD-L1 signaling axis

**FIGURE 9:**
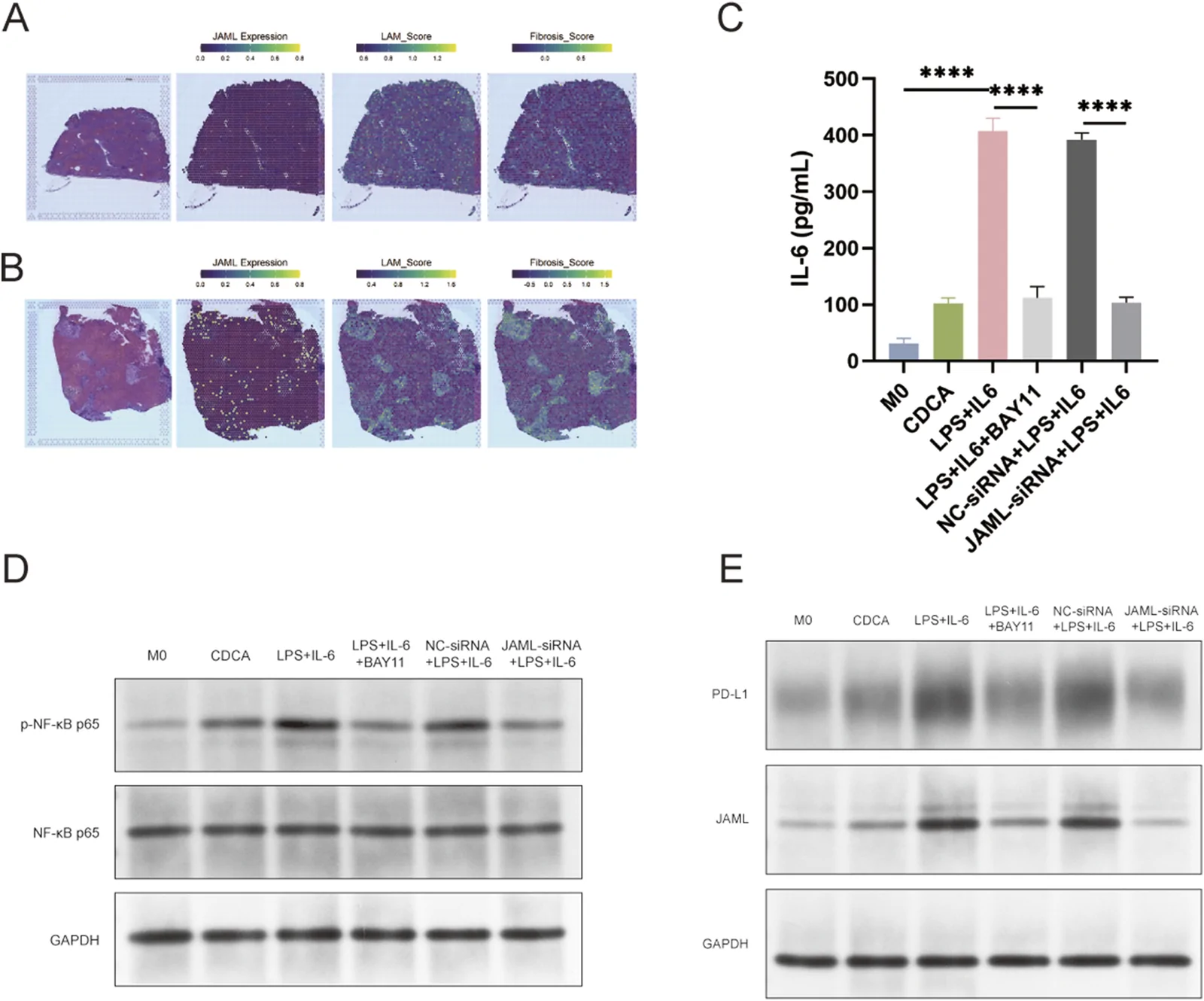
*In vitro* validation of JAML-dependent NF-κB activation, PD-L1 expression, and IL-6 secretion in macrophages. **(A,B)** Representative spatial distribution maps and scoring heatmaps showing the correlation between JAML expression, LAM Score, and Fibrosis Score in tissue sections. Color scales indicate the intensity of expression or severity of scores. **(C)** Concentrations of IL-6 in macrophage culture supernatants measured by ELISA. Macrophages were treated with M0 (control), CDCA, LPS + IL-6, LPS + IL-6+BAY11 (NF-κB inhibitor), NC-siRNA + LPS + IL-6 (negative control siRNA), or JAML-siRNA + LPS + IL-6. Data are presented as mean ± SD. P < 0.0001. **(D)** Western blot analysis of phospho-NF-κB p65 (p-p65), total NF-κB p65, and GAPDH (loading control) in THP-1-derived macrophages under the indicated treatment conditions. **(E)** Western blot analysis of PD-L1, JAML, and GAPDH expression in THP-1-derived macrophages under the indicated treatment conditions.

To further validate the computationally inferred JAML-associated inflammatory phenotype, we integrated spatial transcriptomic analysis with *in vitro* functional experiments. Spatial transcriptomic analysis was performed using four consecutive sections from one PSC liver and one healthy control liver. In PSC sections, regions with higher JAML expression showed spatial overlap with areas exhibiting higher LAM and fibrosis scores, whereas JAML expression was low or absent in control sections without obvious spatial enrichment of LAM or fibrosis scores (Figures 9A,B). Across the four PSC sections, Spearman correlations ranged from 0.064 to 0.120 for JAML vs. LAM score, 0.136 to 0.144 for JAML vs. fibrosis score, and 0.160 to 0.332 for LAM score vs. fibrosis score (all P < 0.001; Additional file 6: Supplementary Figure S4A), while these correlations were not significant in control sections (all P > 0.05; Additional file 6: Supplementary Figure S4B). To further investigate the potential molecular mechanism, PMA-differentiated THP-1 macrophages were stimulated with LPS plus IL-6. ELISA analysis showed that inflammatory stimulation significantly increased IL-6 secretion compared with M0 control or CDCA treatment, whereas NF-κB inhibition by BAY 11–7,082 or JAML knockdown significantly reduced IL-6 production (Figure 9C). Western blot analysis demonstrated that LPS + IL-6 stimulation increased p-NF-κB p65, JAML, and PD-L1 expression, while total p65 levels remained unchanged (Figures 9D,E). Consistently, BAY 11–7,082 treatment attenuated JAML and p-NF-κB induction, and JAML knockdown reduced NF-κB phosphorylation and PD-L1 expression under inflammatory conditions (Figures 9D,E). These findings suggest that JAML is associated with fibrosis-related spatial features in PSC and may functionally participate in maintaining the inflammatory activation state of macrophages through NF-κB-associated signaling. Original, uncropped Western blot images are provided in Additional file 9.

## Discussion

Primary sclerosing cholangitis (PSC) is an immune-mediated chronic cholestatic liver disease characterized by progressive peribiliary inflammation and obliterative fibrosis. No disease-modifying therapy is currently approved, and liver transplantation remains one of the most clearly effective options to improve long-term outcomes (Guicciardi et al., 2018). Accumulating evidence indicates that macrophages occupy a central position in the maintenance of inflammation, tissue remodeling, and fibrosis progression in PSC (Bernard et al., 2023). However, it remains poorly understood how genetic susceptibility relates to specific pathogenic immune states at the levels of cell type and cell state. In this study, we mapped transcriptome-wide association study (TWAS) signals onto a single-cell transcriptomic atlas of PSC livers and found that genetic risk-related transcriptional activity was predominantly enriched in the monocyte/macrophage lineage. This suggests that genetic risk-associated transcriptional activity may be more concentrated in myeloid cells and could contribute to disease progression by influencing their differentiation and functional status, rather than being uniformly distributed across multiple liver cell types.

Beyond fibrosis, PSC is characterized by chronic immune dysregulation, including altered innate and adaptive immune responses, immune checkpoint expression, and cytokine networks (Di Tinco et al., 2026). The observed negative correlation between JAML expression and activated CD8^+^ T-cell infiltration, together with the association between JAML-high LAMs and IL-6/JAK/STAT3 and TNFα/NF-κB signaling, raises the possibility that JAML^+^ LAMs contribute to an immunosuppressive or T-cell-excluded microenvironment. Recent work has linked IL-6 signaling to PD-1/PD-L1 upregulation in cholangiocytes via pSTAT3/NF-κB activation in PSC, suggesting that macrophage-derived IL-6 from JAML^+^ LAMs could promote epithelial immune checkpoint expression and sustain chronic inflammation (Orlandi et al., 2026). Our *in vitro* data showing JAML-dependent IL-6 secretion and PD-L1 upregulation in macrophages are consistent with this hypothesis, although direct macrophage-cholangiocyte co-culture experiments will be required to establish this axis functionally.

Within the monocyte/macrophage lineage, lipid-associated macrophages (LAMs) characterized by TREM2^+^, GPNMB^+^, and APOC1^+^ exhibited the highest TWAS activity and were located at a late stage of pseudotime differentiation trajectories, indicating that genetic risk may exert its effects predominantly in a terminal functional state. This observation is consistent with recent single-cell and spatial mapping studies of PSC livers reporting enrichment of LAM-like macrophages (Cheng et al., 2023), and it also aligns with evidence from cirrhosis showing that TREM2^+^ scar-associated macrophages can shape the fibrotic niche through pro-fibrotic mediators such as TGF-β1 and PDGF (Ramachandran et al., 2019). Based on TWAS composite scores, we further stratified LAMs into high TWAS activity (HTS) and low TWAS activity (LTS) states. HTS cells were enriched in fibrosis-related pathways (e.g., TGF-β, NOTCH, and WNT/β-catenin) as well as innate immune pathways (e.g., TLR2/4-MAPK and the NLRP3 inflammasome), suggesting that HTS cells concurrently display inflammatory and tissue repair/fibrosis-associated functional features that are not readily captured by the traditional M1/M2 dichotomy (Dewidar et al., 2019; Murray and Wynn, 2011). In this framework, macrophages may sustain inflammatory input through innate immune signaling while also promoting hepatic stellate cell (HSC) activation and extracellular matrix (ECM) deposition via axes such as TGF-β, potentially reinforcing peribiliary inflammation and fibrosis. This interpretation is consistent with recently reported macrophage subsets characterized by broadly pro-fibrotic transcriptional programs (Fabre et al., 2023).

Spatial transcriptomics further linked these cellular states to tissue architecture. Data were obtained from four consecutive 10× Genomics Visium FFPE sections from one PSC liver and one healthy control liver (GEO accessions GSE245620 and GSE240429, respectively). In PSC livers, JAML-positive regions overlapped with areas of higher LAM scores and higher fibrosis scores, whereas JAML was nearly absent in normal livers. Although the spatial correlation between JAML and fibrosis score was moderate (ρ ≈ 0.144), this does not necessarily negate biological relevance. Spatial transcriptomic spots often contain multiple cell types, and spatial misalignment or temporal lags between inflammatory infiltration and collagen deposition may attenuate correlation estimates. Moreover, PSC lesions are patchy, and cell composition as well as the degree of tissue remodeling can vary across regions. Consistent with this concept of spatial heterogeneity in fibrotic liver, Watson et al. constructed a single-cell spatial atlas of healthy and fibrotic human livers using MERFISH and demonstrated pronounced spatial remodeling of macrophage and mesenchymal populations in fibrotic tissue (Watson et al., 2025). Therefore, JAML may represent a spatial signature accompanying fibrosis-associated microenvironments, which warrants validation in larger spatial cohorts and functional experiments.

To identify key molecules associated with the HTS phenotype, we applied multi-algorithm feature selection and identified JAML (AMICA1) as the core gene discriminating HTS from LTS cells. Previous studies mainly considered JAML as a co-stimulatory receptor for epithelial γδ T-cell activation (Verdino et al., 2010; Witherden et al., 2010). Recent evidence indicates that JAML can also promote NF-κB signaling and NLRP3 inflammasome activation in macrophages, thereby accelerating atherosclerosis progression (Cui et al., 2025). In an acute kidney injury model, JAML skewed macrophages toward a pro-inflammatory phenotype and suppressed efferocytosis (Huang et al., 2022). In the present study, protein-level validation in LPS/IL-6-stimulated macrophages showed that JAML upregulation accompanied NF-κB p65 phosphorylation and PD-L1 expression, and that JAML knockdown reduced these inflammatory outputs. These observations support the interpretation that JAML participates in a positive feedback loop with NF-κB signaling in activated macrophages, rather than being a passive marker of activation. In the chronic fibrotic microenvironment of PSC examined here, JAML-high LAMs were enriched in pathways related to repair responses and metabolic reprogramming (e.g., TGF-β and PPAR), suggesting that JAML-associated programs may be context-dependent (Murray and Wynn, 2011). In addition, immune infiltration analysis showed that JAML expression was negatively correlated with activated CD8^+^ T-cell abundance, implying a potential association with an immunosuppressive or T-cell-excluded microenvironment. This contrasts with findings in lung cancer, where JAML is highly expressed in CD8^+^ T cells and enhances anti-tumor immunity through binding to CXADR (Hao et al., 2024), highlighting that JAML may have distinct cellular sources and immunological roles across disease contexts.

Virtual knockout analysis using scTenifoldKnk suggested that JAML may influence MHC class II antigen presentation, collagen-containing ECM remodeling, and endosomal/lysosomal functions. Together with evidence that activated HSCs upregulate antigen-presenting molecules (Maubach et al., 2007) and that lysosomal dysfunction can induce HSC activation and promote fibrosis (Lee et al., 2024), we hypothesize that JAML + LAMs may indirectly facilitate HSC activation and fibrosis progression through processes involving antigen presentation, lysosomal function, and ECM remodeling. Notably, virtual knockout results primarily reflect network perturbation strength rather than regulatory direction; therefore, these inferences require validation by directional and causal *in vivo* and *in vitro* experiments. Cell–cell communication analysis further showed that JAML-positive LAMs exhibited enhanced incoming and outgoing signals in pathways including MIF, VEGF, complement, GALECTIN, and SPP1. MIF regulates innate immune responses and sustains inflammation (Calandra and Roger, 2003); VEGF is associated with peribiliary vascular plexus remodeling and cholangiocyte proliferation in cholestatic liver disease (Mariotti et al., 2021); and complement activation may contribute to cholestatic liver injury by modulating macrophage infiltration and function (Guo et al., 2021). Of note, SPP1 is a marker of bile duct-associated macrophages and correlates with fibrosis severity in PSC (De Muynck et al., 2024), and aberrant GALECTIN signaling has been proposed to contribute to inflammation and fibrosis in PSC (Grewal et al., 2025). Collectively, these results suggest that JAML + LAMs are integrated into the pro-fibrotic microenvironment of PSC through paracrine interactions with cholangiocytes, HSCs, and other immune cells. Combined with the dynamic changes in JAML expression along pseudotime, we further postulate that JAML may be involved in a transition phase during which LAMs acquire a highly active state. Time-resolved blockade experiments (e.g., neutralizing antibodies or CRISPR interference) in vitro macrophage differentiation systems may help test whether targeting JAML can alter the trajectory toward a pro-fibrotic phenotype.

Several limitations should be acknowledged. First, the scRNA-seq atlas is derived primarily from end-stage PSC livers undergoing transplantation, and the low representation of hepatocytes, cholangiocytes, and mesenchymal cells reflects the advanced cirrhotic stage of the samples rather than early disease events. This may bias TWAS enrichment analyses toward immune-cell-biased signals and limits inferences about disease initiation. Second, feature selection for HTS versus LTS cells was primarily based on internal data, and external single-cell cohorts for independent validation are currently limited. Third, spatial transcriptomics was performed on serial sections from one PSC patient and one healthy control (four sections each), without patient-level biological replicates; therefore, the generalizability of the observed spatial patterns requires further confirmation. Fourth, virtual knockout analysis cannot establish regulatory directionality or causality. Fifth, the *in vitro* validation used a macrophage cell-line model and focused on protein readouts in macrophages; it does not capture epithelial-macrophage crosstalk, adaptive immune components, or the full spatial heterogeneity of PSC liver. Sixth, although bulk RNA-seq-based immune infiltration analysis showed that JAML expression was associated with neutrophils and plasmacytoid dendritic cells, these low-abundance immune populations were not sufficiently represented to form distinct clusters in our scRNA-seq atlas. Therefore, the expression pattern and potential functional role of JAML in these immune subsets could not be reliably evaluated in the current dataset. Future studies using larger single-cell cohorts or targeted immune profiling approaches are needed to further investigate JAML functions beyond macrophage populations. Future studies combining PSC animal models (e.g., DDC-fed mouse models) with macrophage-specific JAML conditional knockout, neutralizing antibodies, macrophage-cholangiocyte Transwell co-culture, and multi-patient spatial cohorts will be important to directly test the functional role of JAML in peribiliary inflammation and fibrosis and to evaluate its translational potential.

Taken together, we propose a working hypothesis. Genetic risk-related transcriptional activity in PSC is mainly concentrated in the monocyte/macrophage lineage and is most prominent in the terminal functional state of LAMs. JAML + LAMs exhibit higher TWAS activity, a fibrosis-associated transcriptional signature, enhanced cell–cell communication signals, and spatial co-localization with fibrotic areas. These findings suggest that JAML + LAMs may represent an effector cell population linking genetic susceptibility to peribiliary fibrosis, providing a testable direction for mechanistic studies of PSC and for interventions targeting myeloid cell states.

## Data Availability

The original contributions presented in the study are included in the article/Supplementary Material. The datasets analyzed in this study can be found in online repositories, and the accession numbers are provided in the article. Further inquiries can be directed to the corresponding authors.
