# Supplementary material for: Multi-omics mapping of PSC genetic risk to a high TWAS-active JAML+ lipid-associated macrophage program: bridging single-cell heterogeneity, spatial fibrotic niches, and machine learning selection
Source: Front Cell Dev Biol. 2026 Sep 4;14:1923026. doi: 10.3389/fcell.2026.1923026 (PMC13548120; doi:10.3389/fcell.2026.1923026)
Supplement: Supplementary file 1 [file DataSheet2.pdf]

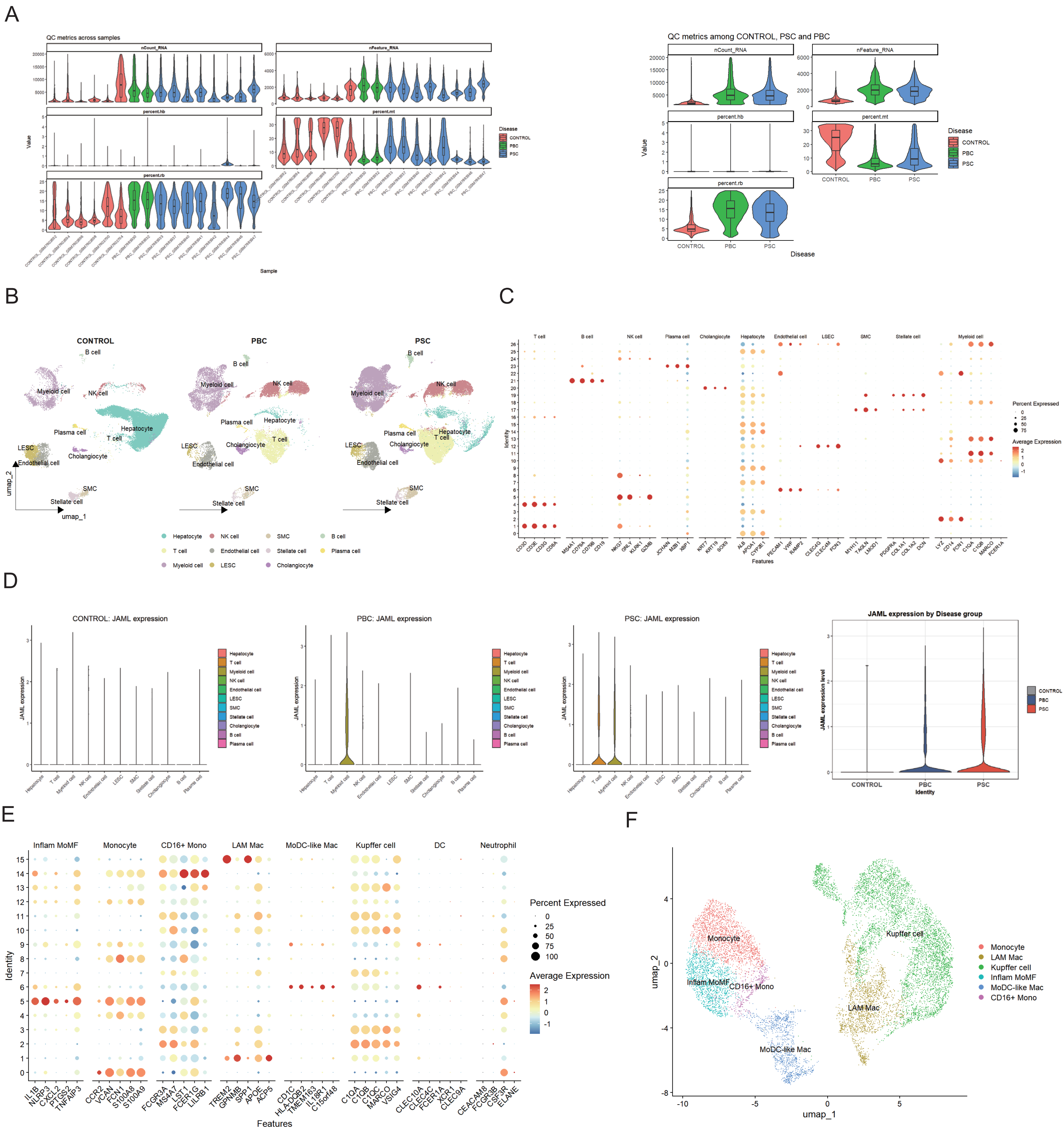

Additional file 2: Figure S1. Single-cell quality control, global atlas, JAML expression, and myeloid sub-clustering. (A) Quality control metrics across individual samples (left) and among disease groups (right), showing nFeature_RNA, nCount_RNA, percent.mt, percent.rb, and percent.hb. (B) UMAP embedding of all cells colored by cell type annotation, split by disease group (CONTROL, PBC, PSC). (C) Dot plot showing expression of canonical marker genes across the 27 global clusters. (D) Violin plots showing JAML expression by disease group (left) and by cell type (right). (E) UMAP embedding of re-clustered myeloid subsets. (F) Dot plot showing marker gene expression across re-clustered myeloid subsets.
