# Supplementary material for: Multi-omics mapping of PSC genetic risk to a high TWAS-active JAML+ lipid-associated macrophage program: bridging single-cell heterogeneity, spatial fibrotic niches, and machine learning selection
Source: Front Cell Dev Biol. 2026 Sep 4;14:1923026. doi: 10.3389/fcell.2026.1923026 (PMC13548120; doi:10.3389/fcell.2026.1923026)
Supplement: Supplementary file 2 [file DataSheet3.pdf]

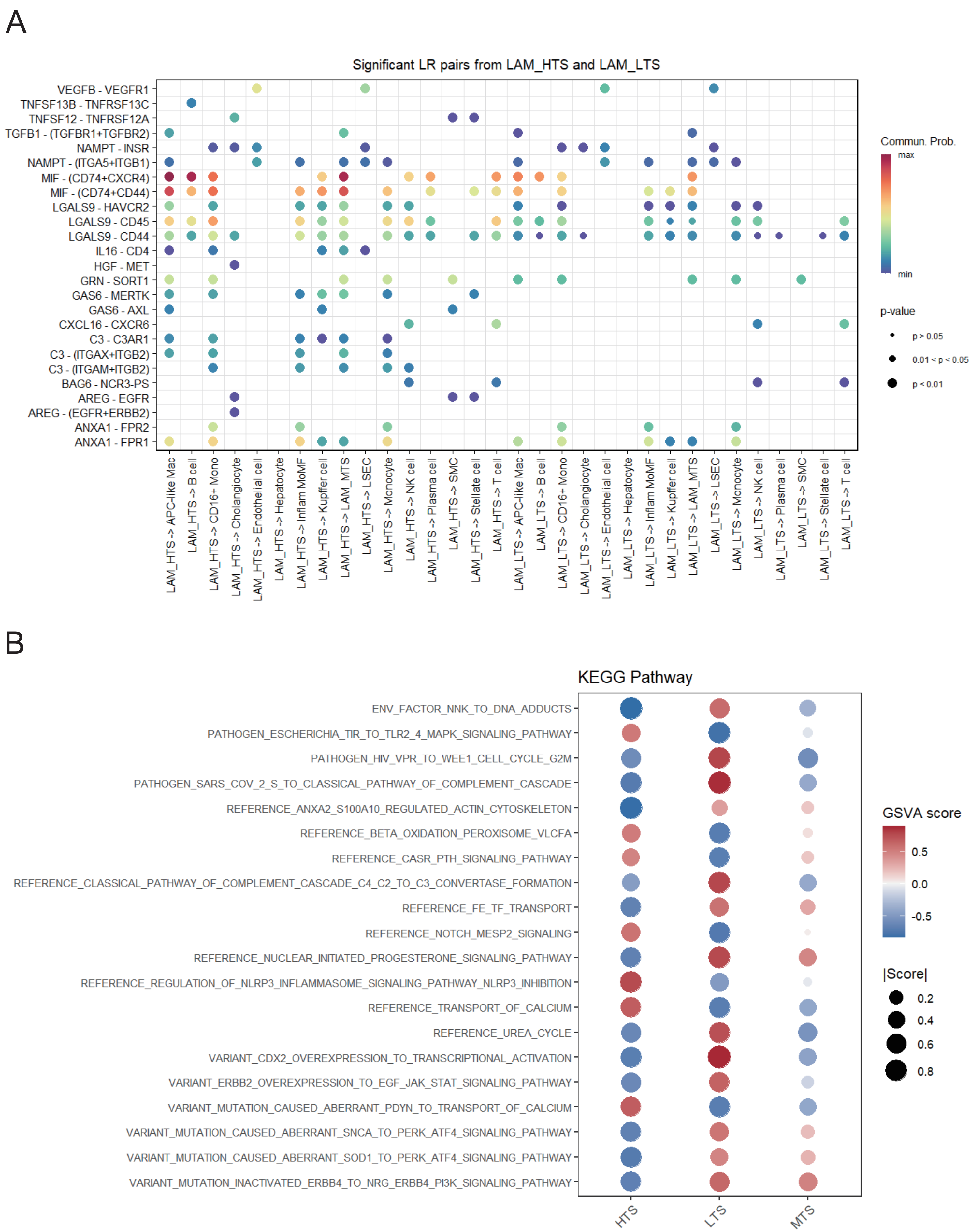

Additional file 3: Figure S2. Supplementary pathway analyses of LAM subsets. (A) Ligand–receptor interaction network of the MIF pathway between LAM_HTS and other cell types. (B) KEGG_MEDICUS-based GSVA heatmap comparing HTS, MTS, and LTS groups.
