# Supplementary material for: Multi-omics mapping of PSC genetic risk to a high TWAS-active JAML+ lipid-associated macrophage program: bridging single-cell heterogeneity, spatial fibrotic niches, and machine learning selection
Source: Front Cell Dev Biol. 2026 Sep 4;14:1923026. doi: 10.3389/fcell.2026.1923026 (PMC13548120; doi:10.3389/fcell.2026.1923026)
Supplement: Supplementary file 3 [file DataSheet5.pdf]

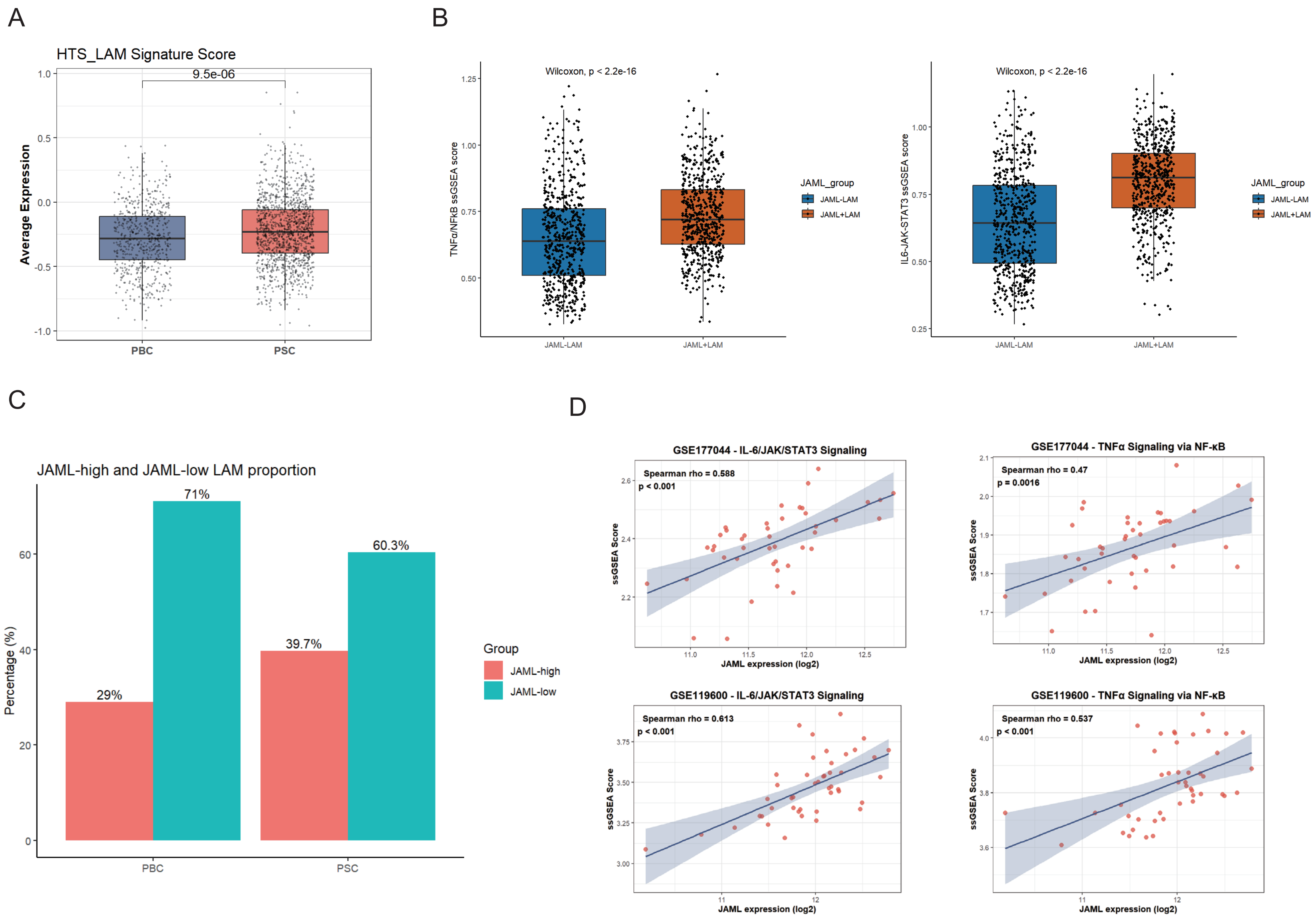

Additional file 5: Figure S3. Association of JAML expression with LAM activation states and inflammatory pathway activity. (A) Boxplot of LAM module score derived from the 7 core HTS signature genes (JAML, TACC1, C1orf43, GLIPR1, ALDH2, SLCO2B1, and ARHGIP4) comparing PBC and PSC LAMs. (B) Boxplots of TNFα/NF-κB and IL-6/JAK/STAT3 pathway activity (ssGSEA scores) comparing JAML+ and JAML- LAMs. (C) Bar plot showing the proportion of JAML-high and JAML-low LAMs in PSC versus PBC. (D) Correlation scatter plots of JAML expression versus TNFα/NF-κB and IL-6/JAK/STAT3 pathway ssGSEA scores in bulk RNA-seq datasets GSE119600 and GSE177044.
