# Supplementary material for: Multi-omics mapping of PSC genetic risk to a high TWAS-active JAML+ lipid-associated macrophage program: bridging single-cell heterogeneity, spatial fibrotic niches, and machine learning selection
Source: Front Cell Dev Biol. 2026 Sep 4;14:1923026. doi: 10.3389/fcell.2026.1923026 (PMC13548120; doi:10.3389/fcell.2026.1923026)
Supplement: Supplementary file 4 [file DataSheet6.pdf]

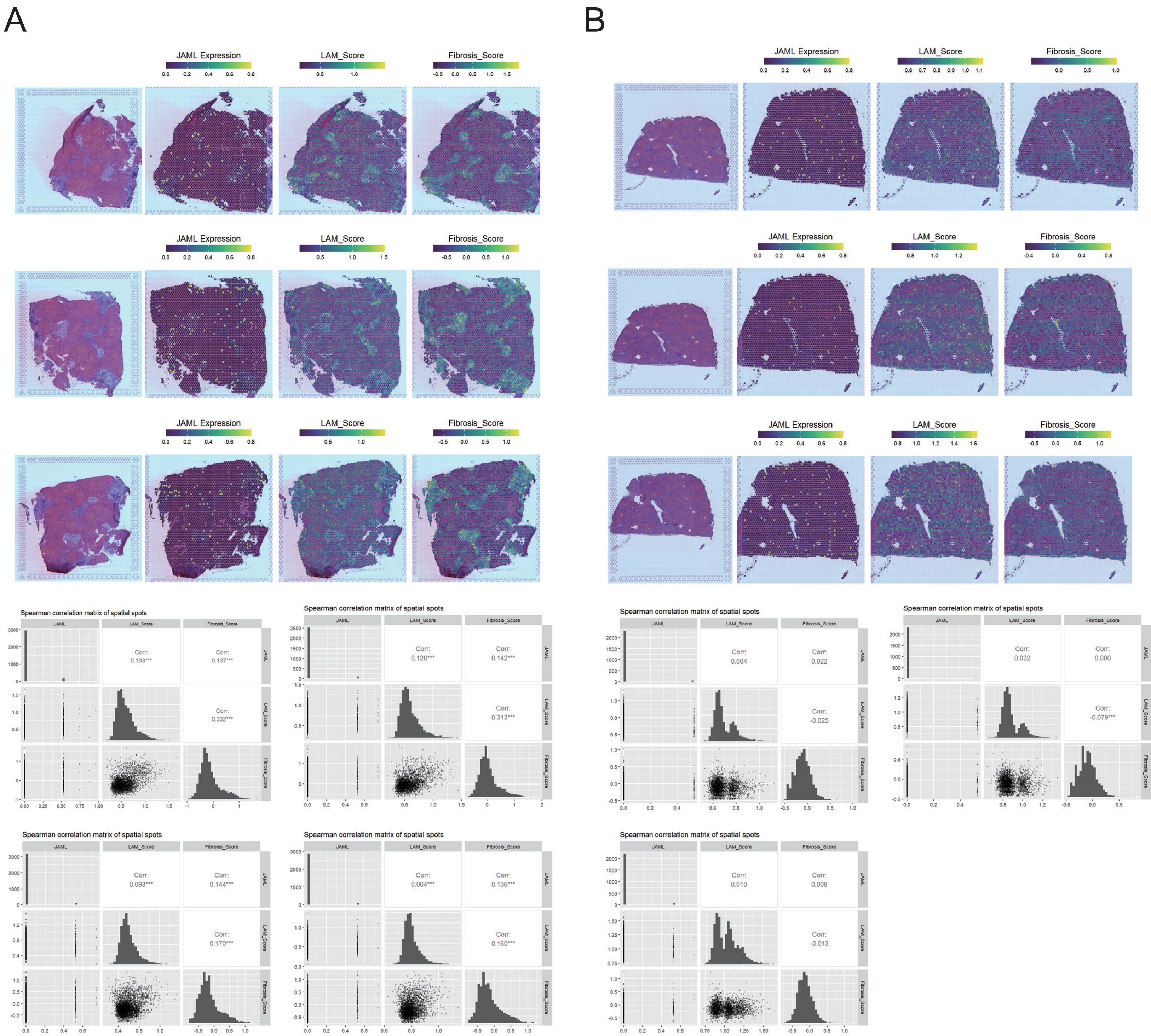

Additional file 6: Figure S4. Spatial transcriptomics correlation analyses. (A) Spearman correlations between JAML, LAM score, and fibrosis score across four consecutive tissue sections from a single PSC patient (GSE245620). (B) Corresponding correlations across four consecutive tissue sections from a single healthy control (GSE240429).
