# Supplementary material for: Multi-omics mapping of PSC genetic risk to a high TWAS-active JAML+ lipid-associated macrophage program: bridging single-cell heterogeneity, spatial fibrotic niches, and machine learning selection
Source: Front Cell Dev Biol. 2026 Sep 4;14:1923026. doi: 10.3389/fcell.2026.1923026 (PMC13548120; doi:10.3389/fcell.2026.1923026)

GAPDH

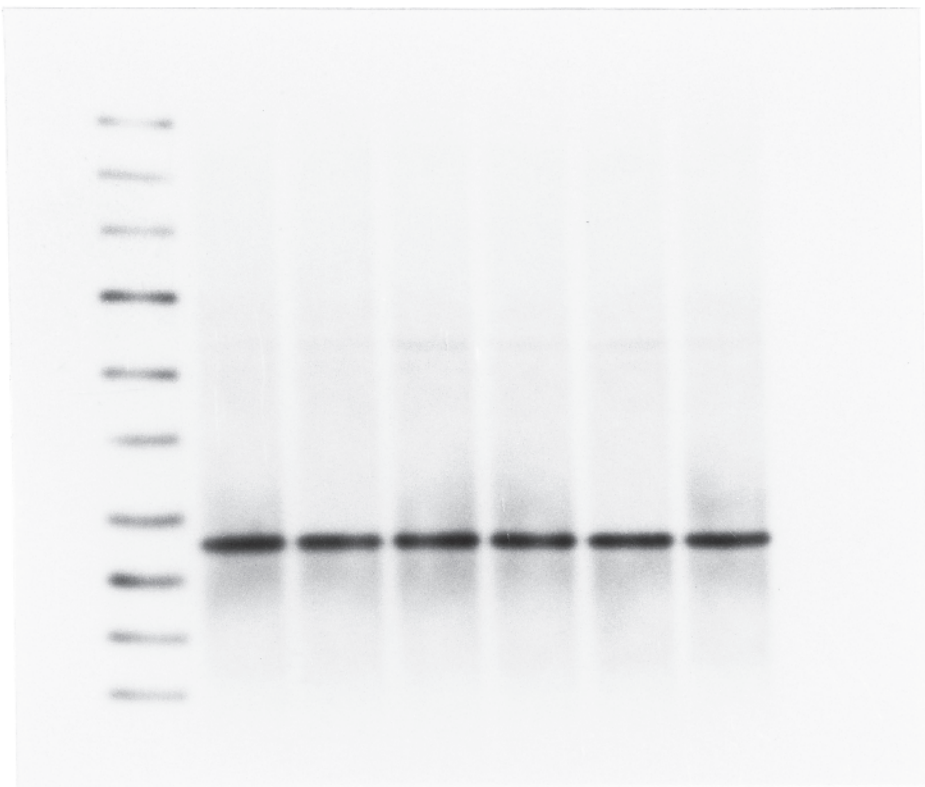

NF-κB p65 (Total)

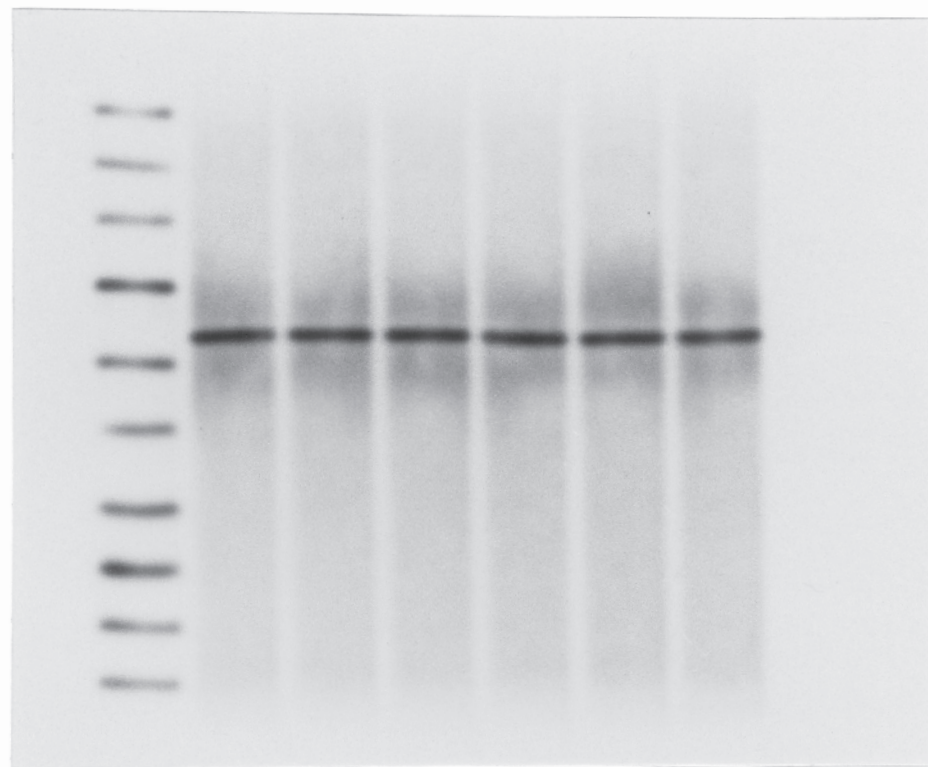

p-NF-κB p65 (Ser536)

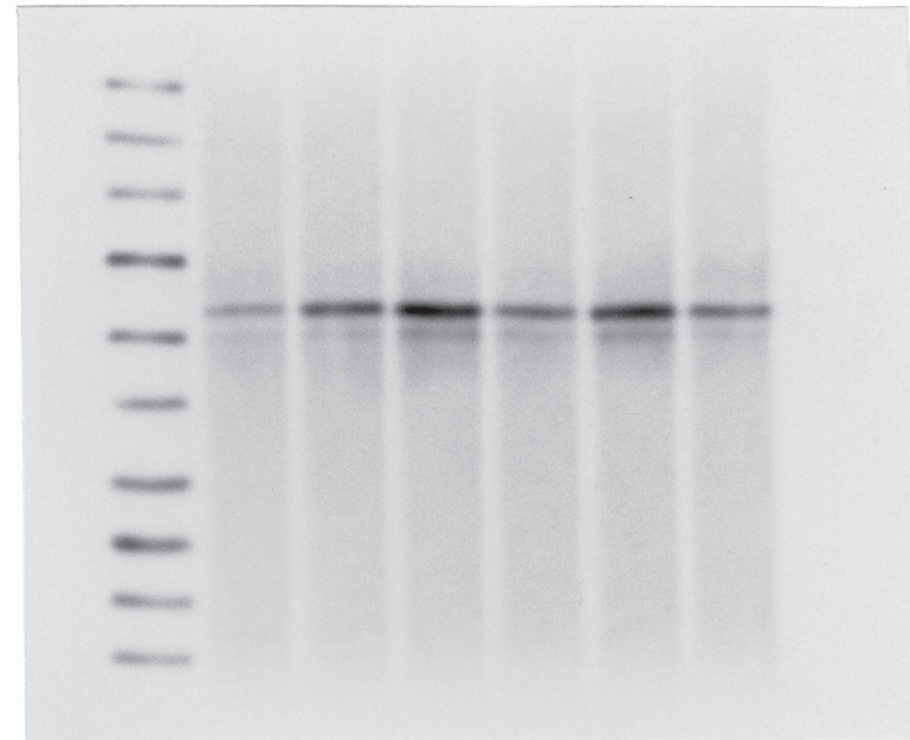

GAPDH

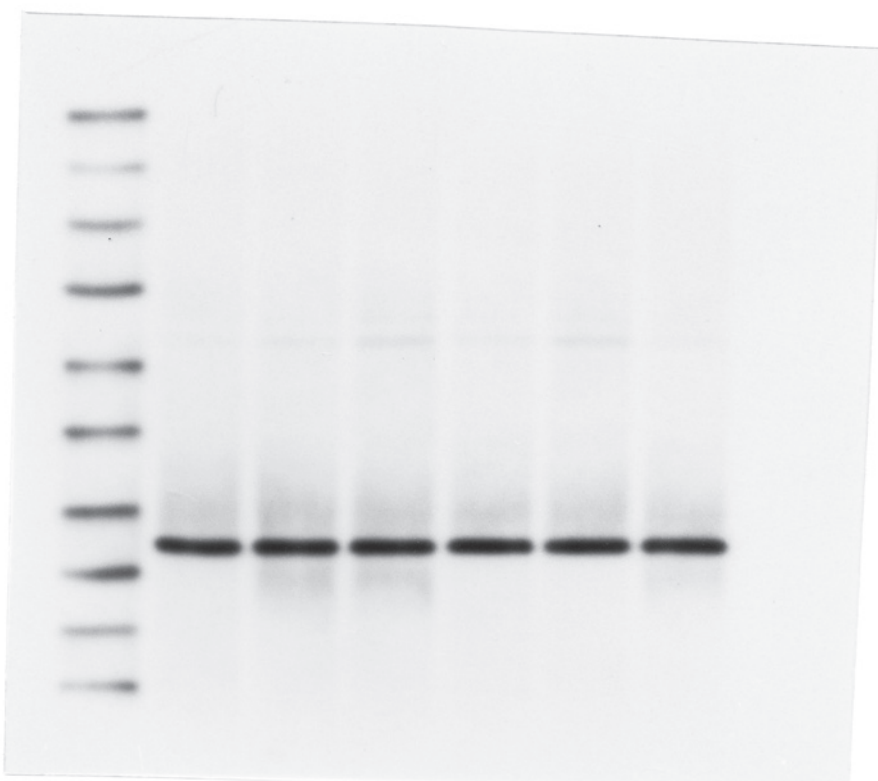

JAML

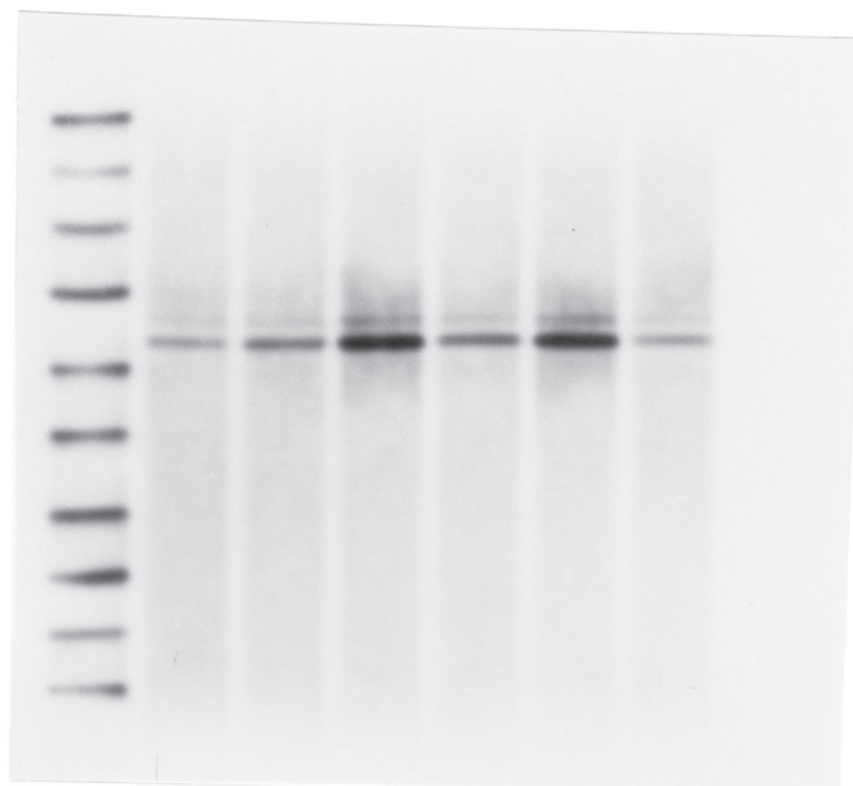

PD-L1 (CD274)

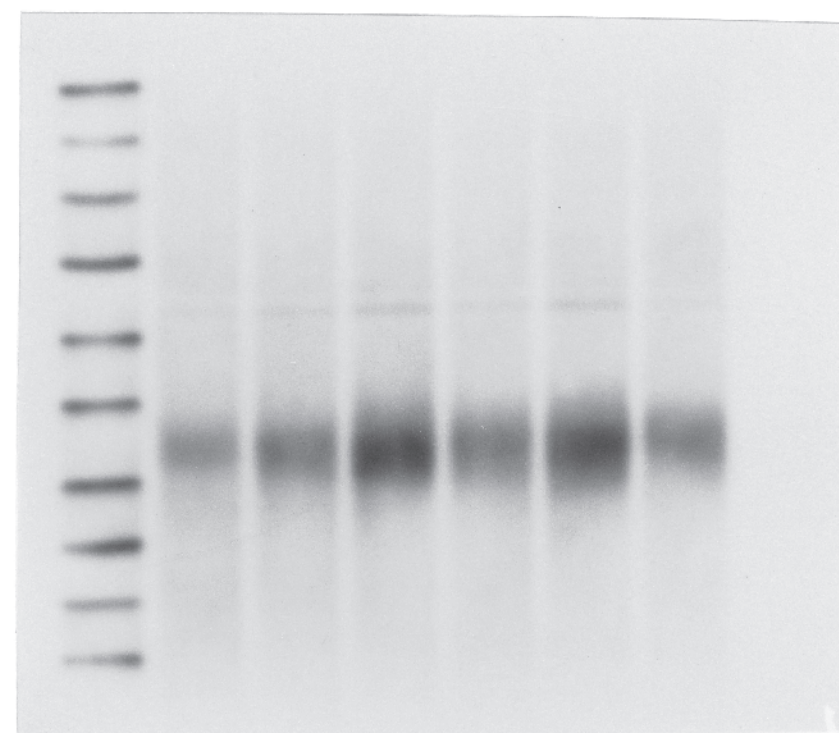

Supplement: Supplementary file 5 [file DataSheet9.pdf]
